# Evidence for Transient Ylide Intermediates of the Type ^t^Bu_2_P(H)=CHZ That Are Prototropic Tautomers of ^t^Bu_2_PCH_2_Z

**DOI:** 10.1021/acs.inorgchem.6c02065

**Published:** 2026-06-29

**Authors:** E. Louise Hazeland, Chen Li, Nicholas P. Taylor, Guy C. Lloyd-Jones, Paul G. Pringle

**Affiliations:** † School of Chemistry, University of Bristol, Cantocks Close, Bristol BS8 1TS, U.K.; ‡ School of Chemistry, 3124 University of Edinburgh, Joseph Black Building, David Brewster Road, Edinburgh EH9 3FJ, U.K.

## Abstract

The mechanism of the formation of ^t^Bu_2_PCH_2_P­(1,1′-bi-2-naphtholate) (**3**) from the P–Si exchange reaction between ^t^Bu_2_PCH_2_SiMe_3_ (**1**) and ClP­(1,1′-bi-2-naphtholate) (**2**) is explored. Through an iterative process of kinetic measurements and mechanistic hypotheses, the evidence supports the intermediacy of the ylide ^t^Bu_2_PH=CHSiMe_3_. The reaction of **1** with **2** is efficiently catalyzed by HCl. In the absence of **2**, HCl reacts with **1** to give [^t^Bu_2_PHCH_3_]­Cl, and it is suggested that this protodesilylation proceeds via the ylide ^t^Bu_2_PH=CH_2_ (**Y1**), the prototropic tautomer of ^t^Bu_2_PCH_3_. The products observed upon treatment of **1** with DCl, support the formation of ^t^Bu_2_PD=CH_2_ (*d-* **Y1**) but, in addition, they support the reversible formation of ^t^Bu_2_PD=CHSiMe_3_ (*d-* **Y2**). When **3** was treated with the chlorophosphite ClP­(3,3′-diphenyl-1,1′-bi-2-naphtholate) (**2a**), an equilibrium mixture of **2**, **2a**, **3** and ^t^Bu_2_PCH_2_P­(3,3′-diphenyl-1,1′-bi-2-naphtholate) (**3a**) is formed. Kinetic modeling of the proposed mechanism of the formation of **3** from **1** and **2** via the ylide intermediate **Y2** correlates well with the experimental data obtained under a variety of conditions and provides strong support for the veracity of the mechanism.

## Graphical abstract

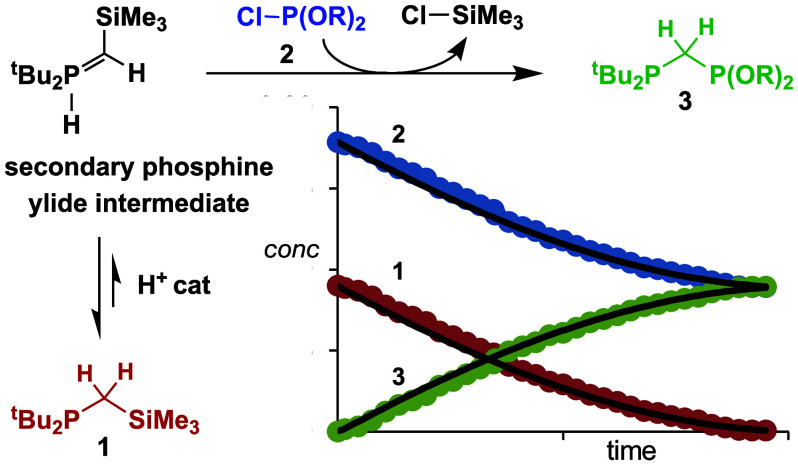

## Introduction

Prototropism is well established in secondary phosphine oxides with the position of the equilibrium (eq 1, Z = O) determined by the basicity of the R_2_P group and the acidity of the OH group.^1–4^ In almost every case, the P­(V) isomer is the major tautomer,^5,6^ with a notable exception being for R = CF_3_, when the hydroxyphosphine P­(III) tautomer is favored due to the very weak basicity of the P­(CF_3_)_2_ group.^7^ Similar considerations apply to secondary phosphine amides (eq 1, Z = NR′) but the lower electronegativity of N than O leads to a shift in stability toward the aminophosphine P­(III) tautomer.^1,8^ When it comes to tertiary phosphines (eq 1, Z = CR′_2_), the P­(III) tautomer is overwhelmingly preferred.^9^ Exceptions arise when the R′ substituents are highly electron-withdrawing; e.g., when Z = C­(CO_2_Me)_2_ or C­(SO_2_Tol)_2_, the tautomeric equilibria are finely balanced.^1,10–14^


1

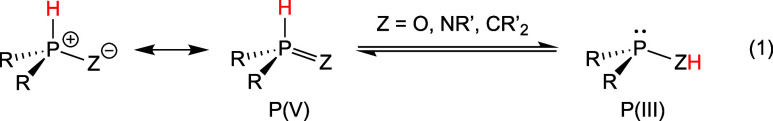

 In this article, we present evidence for the involvement of P­(V) ylide tautomers of the type ^t^Bu_2_PH=CHZ as intermediate in the P–Si exchange reaction shown in eq 2, in which phosphine **1** and chlorophosphite **2** give the *C*_1_-symmetric, C_1_-backboned ligand **3** previously reported.^15,16^


2

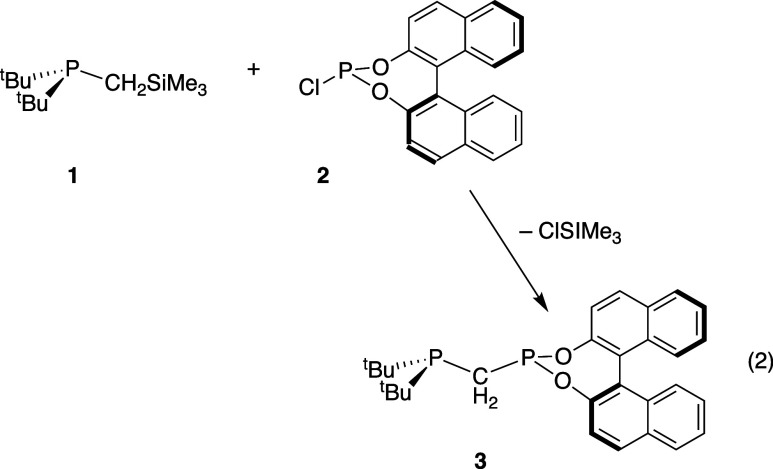




## Results and Discussion

### Initial Kinetic Observations

The reaction shown in eq 2 was monitored by ^31^P­{^1^H} NMR spectroscopy, and the only signals observed were the P-containing reactants **1** and **2** and product **3** with no intermediates and no other P-containing products detected. A ^29^Si­{^1^H} NMR signal was observed at δ 31.3 ppm, consistent with the formation of Me_3_SiCl as byproduct which was corroborated by a ^1^H NMR signal at 0.42 ppm which is close to the literature value for Me_3_SiCl.^17,18^ No reaction took place when the borane adduct ^t^Bu_2_P­(BH_3_)­CH_2_SiMe_3_ (**1**·BH_3_) was treated with chlorophosphite **2**, indicating that the lone pair on **1** is essential for the formation of the diphos **3**. The reaction was noticeably slower in toluene than in the more polar THF; this is clear from a comparison of the kinetic plots in Figures S1 and S2 (see the SI) for the reaction carried out in toluene and THF, under otherwise similar conditions. Qualitative rates of the reactions, as measured by the time taken to be ca. 50% complete, depended on the R_2_P group in R_2_PCH_2_SiMe_3_: the rate was faster when R_2_P = Cy_2_P than when R_2_P = Ph_2_P, and faster when R_2_P = ^i^Pr_2_P than when R_2_P = ^t^Bu_2_P, indicating that the rate depended on the basicity and steric accessibility of the lone pair on P.

The mechanism shown in Scheme 1, was initially proposed as it is consistent with the above observations. Thus, nucleophilic attack by the phosphine **1** on chlorophosphite **2** to give a P–P bonded ionic intermediate **A** which then undergoes a chloride-induced desilylation to give ylide **B**, followed by an intramolecular rearrangement to give the observed product **3**.

**Scheme 1:**
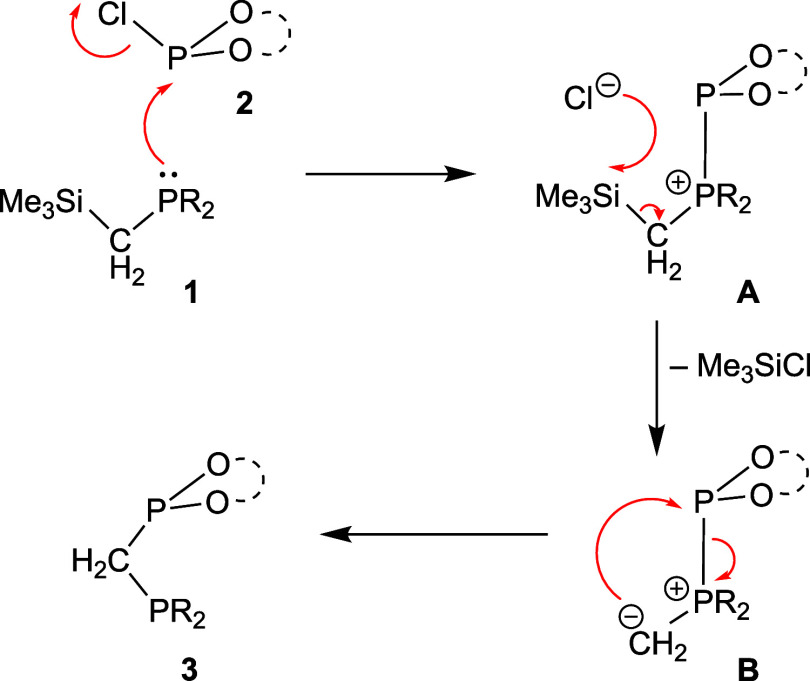
Initial Mechanistic Hypothesis^a^

a The cyclic O,O substituent on P is a doubly-deprotonated 1,1′-bi-2-naphthol.

From the mechanism proposed in Scheme 1, the rate law for a system where neither intermediate (**A** or **B**) accumulates to a significant extent is predicted to be overall second order, being first order with respect to both [**1**] and [**2**]. A kinetic study was carried out on the formation of diphos **3** from phosphine **1** and chlorophosphite **2** in toluene, as this reaction proceeds at a convenient rate to be followed in situ by ^31^P­{^1^H} NMR spectroscopy. The spectra were obtained in such a way that the concentrations of the reactants (**1** and **2**) and product (**3**) could be determined by integration of the appropriate phosphorus peaks. The plots for each of the kinetic runs show an essentially linear decay of both reagents for the bulk of the reaction (Figure 1), with curvature only evident toward the end of the reaction. These plots indicate an initial pseudo-zero-order dependence on both reagents. This conclusion is reinforced by the poor fit of semilogarithmic plots when a 3-fold excess of **1** or of **2** is employed (see Figures S3 and S4).

**Figure 1:**
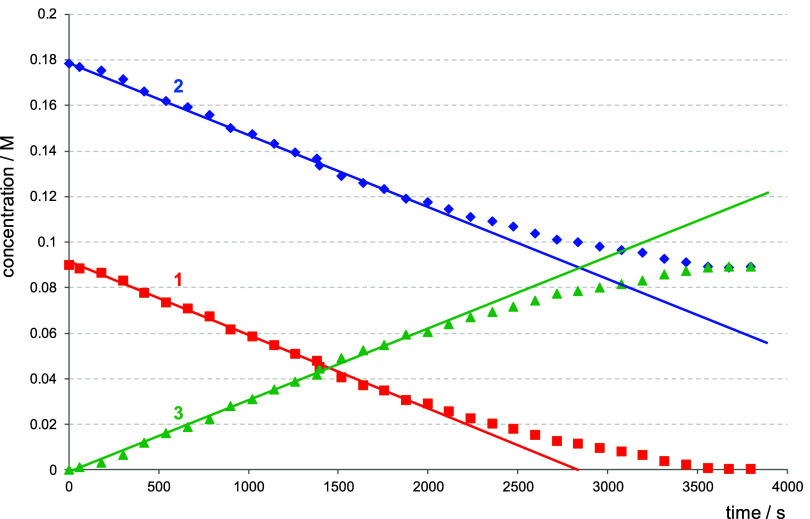
Concentration vs time plots for reactants ^t^Bu_2_PCH_2_SiMe_3_ (**1**) and chlorophosphite **2** in an initial 1:2 molar ratio, and the product diphos **3**, with the straight lines drawn to indicate the initially pseudo-zero-order kinetics.

### Role of Hydrogen Chloride

The initial rates of reaction were determined from the concentration vs. time plots and their values plotted against the initial concentration of each of the reagents. No correlation was observed for the phosphinosilane reagent but, as shown in Figure 2, it is clear that there is a first-order dependence on the *initial* concentration of the chlorophosphite **2**. Since the rate is pseudo-zero-order in [**2**] (see Figure 1), it was inferred that there is a species (**X**) present in the chlorophosphite reagent that is responsible for catalyzing the P–Si exchange reaction.

**Figure 2:**
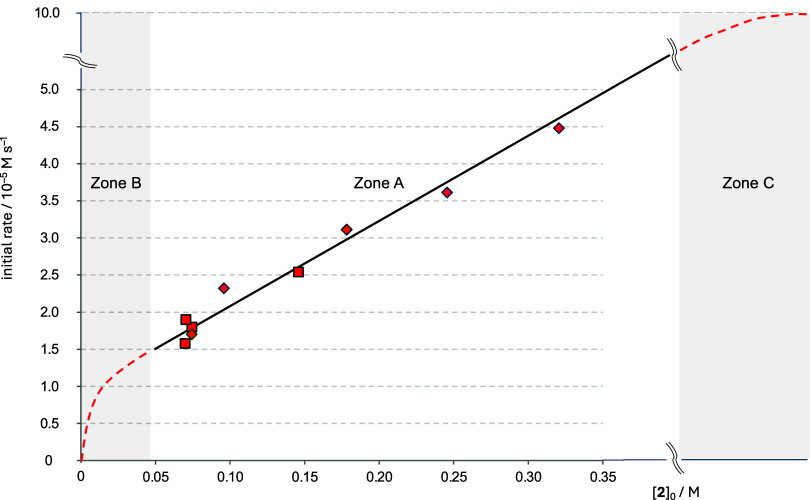
Plot of initial rate vs initial concentration of chlorophosphite ([**2**]_0_). The plot shows a linear increase in rate with increase in [**2** + catalyst **X**]_0_ in Zone A where the rate is pseudo-zero-order in [**2**] (as depicted in Figure 1). Extrapolation of the curve to zero in Zone B, and extrapolation to a limiting rate of approximately 10^–4^ M s^–1^ in Zone C due to saturation in [catalyst **X**] (see Figure 3). The data points indicated by red squares are for experiments where **1** was present in excess, and the red diamonds where **2** was present in excess. The line through the data is a visual guide.

The purity of the chlorophosphite was high (>99%) according to its ^1^H and ^31^P­{^1^H} NMR spectra and therefore either the species **X** has a significant effect even at low concentrations or it contains neither H nor P. Several candidates for **X** were considered (listed in Table 1), and each was added in significant quantities to the P–Si exchange reaction mixture, and the initial rates of reaction measured. The rates (Table 1) when compared with no additive (Entry 1) show: (a) LiCl (Entry 2) or tetra-butylammonium chloride (Entry 3) produced no rate enhancement, ruling out the possibility of chloride as the catalyst **X**; (b) addition of anhydrous HCl (Entry 4) or anhydrous HBF_4_ (Entry 5) produced a significant increase in the initial rate, signaling that H^+^ is a catalyst; addition of water, which would generate HCl by hydrolysis of the chlorophosphite (eq 3), also led to an increase in rate (Entry 6); (c) the addition of Et_3_N (Entry 7) completely inhibited the reaction, which reinforces the conclusion that H^+^ is likely the catalyst. On the basis of these observations, it was proposed the catalytic species **X** present in the chlorophosphite was most likely HCl derived from adventitious H_2_O (eq 3).
3

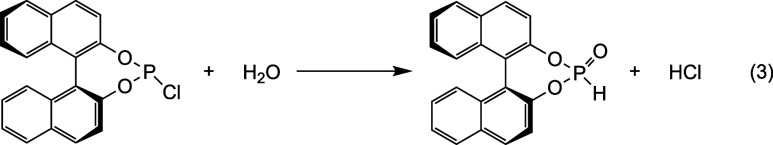




**Table 1:** Initial Rate for P–Si Reaction as a Function of Additive

entry	additive	mol %	initial rate/10^–5^ M s^–1^
1	none	–	3.7
2	LiCl	40.0	3.0
3	[^n^Bu_4_N]Cl	10.0	3.1
4	HCl (Et_2_O)	5.0	9.4
5	HBF_4_ (Et_2_O)	10.0	13.9
6	H_2_O	3.5	7.9
7	NEt_3_	1.0	0.0

Although a rate enhancement was observed from the addition of HCl, the expected first-order dependence on [HCl] was not observed but rather a curve, which demonstrated saturation-like behavior (Figure 3), with the saturation point at low concentrations (<1 mol %). An explanation for this unexpected behavior emerged from the observation of a white solid precipitate upon addition of HCl to the P–Si exchange reaction mixture. The precipitate was identified from its ^31^P (δ_P_ 29.0, ^1^ *J*_PH_ = 490 Hz) and ^1^H NMR spectra in CD_3_CN as the phosphonium salt [^t^Bu_2_P­(H)­CH_2_SiMe_3_]Cl (**1**·HCl). The limited solubility of **1**·HCl in the reaction medium buffers the maximum [HCl] in solution and thereby the HCl-induced rate enhancement. In support of this proposal, at levels of HCl exceeding 2 mol %, a decrease in the absolute integral of the phosphinosilane **1** relative to that of the chlorophosphite **2** was observed (see Figures S7, S1(1)–S1(9)). The calculated amounts of precipitated **1**·HCl from the ^31^P NMR integrals correspond to a [HCl]_0_ = 0.015 M which agrees well with the average value calculated from Figure 4 of [HCl]_0_ = 0.014 M.

**Figure 3:**
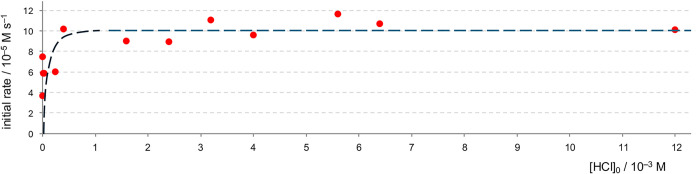
Initial rate vs initial concentration of HCl with the horizontal line at 10^–4^ M s^–1^ representing the apparent saturation rate. The line through the data is a visual guide.

**Figure 4:**
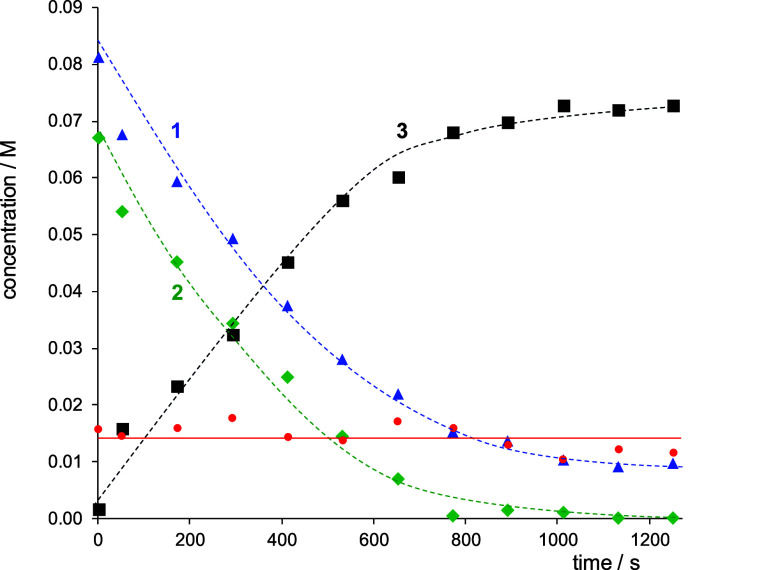
Kinetic profile for the reaction of ^t^Bu_2_PCH_2_SiMe_3_ (**1**) with chlorophosphite **2** to give diphos **3**, in the presence of 15 mol % of HCl (in Et_2_O). The reaction was initiated with [**1**]_0_ = 0.084 M and [**2**]_0_ = 0.083 M. The red circles are the calculated values of ([**2**]_0_ – [**2**]) – ([**1**]_0_ – [**1**]) at each time point; the consistency of the value of *ca*. 0.015 M is due to the buffering effect of the precipitated **1**·HCl on the [HCl] in solution (see Discussion). The lines through the data points are a visual guide.

### Mechanism of the P–Si Exchange Reaction Involving a Secondary Phosphine Ylide

The results from the kinetic study above led to the second proposed mechanism shown in Scheme 2, where the resting state is the phosphonium salt **1**·HCl. The turnover-limiting step (TLS) is ClSiMe_3_ elimination from **1**·HCl to give the secondary phosphine ylide intermediate **Y1**. Ylide **Y1** is a prototropic tautomer of tertiary phosphine ^t^Bu_2_PCH_3_. There is no precedent for the desilylation of the tertiary phosphonium salt **1**·HCl, but Miller reported that vacuum thermolysis of the quaternary phosphonium salt [Me_3_PCH_2_SiMe_3_]Cl resulted in the elimination of ClSiMe_3_ to give the tertiary phosphine ylide Me_3_P=CH_2_ as an intermediate to the ultimate ylide product Me_3_P=CHSiMe_3_.^19^

**Scheme 2:**
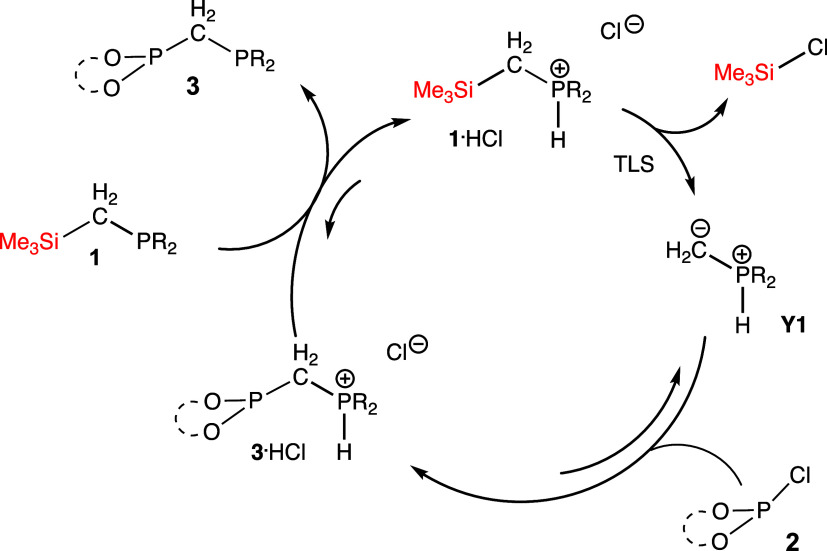
Proposed Mechanism Involving the Secondary Phosphine Ylide **Y1** as an Intermediate

The possibility of ^t^Bu_2_PCH_3_ being in equilibrium with ylide **Y1** was investigated by determining whether isotopic exchange occurs when DCl is added to ^t^Bu_2_PCH_3_ or when HCl is added to ^t^Bu_2_PCD_3_. In neither experiment was D-exchange observed by ^1^H, ^2^H, or ^31^P­{^1^H} NMR spectroscopy, even after 7 days. Furthermore, addition of ^t^Bu_2_PCH_3_ to the chlorophosphite did not lead to diphos **3** formation. These observations suggest that the ClSiMe_3_ loss is integral to the formation of reactive ylide intermediate **Y1**.

When one equivalent of an ethereal solution of HCl was added to phosphinosilane **1** in THF, the ^31^P­{^1^H} NMR spectrum of the reaction mixture showed two singlets at *ca*. 29 and 30 ppm. These were assigned, respectively, to **1**·HCl and the desilylated product [^t^Bu_2_P­(H)­CH_3_]­Cl. Crystals of [^t^Bu_2_P­(H)­CH_3_]Cl were obtained, which were shown to be the same as those for which the X-ray crystal structure has been previously reported.^20^ A mechanism for the protodesilylation of **1** is proposed in Scheme 3 involving ylide **Y1** as an intermediate.

**Scheme 3:**
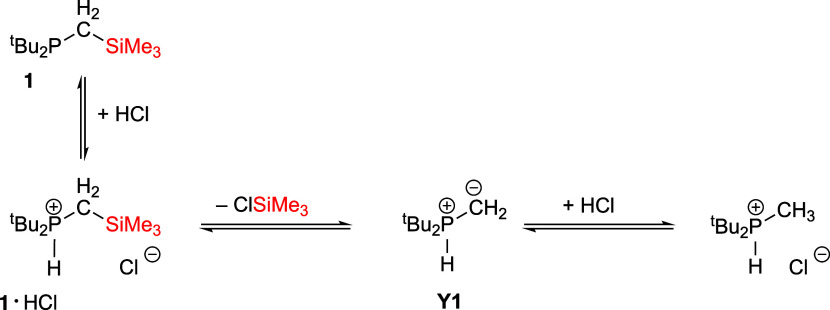
Proposed Mechanism for Protodesilylation of Compound **1** via Ylide Intermediate **Y1**

Addition of 1 equiv of an ethereal solution of DCl to a THF solution of phosphinosilane **1** gave, after 24 h, a mixture of products that have been identified from the ^31^P­{^1^H} NMR spectrum (see Figure S8). In addition to **1** and **1**·DCl (^1^ *J*_PD_ = 145 Hz), the main products are the desilylated species ^t^Bu_2_PCH_2_D and [^t^Bu_2_P­(D)­CH_2_D]­Cl, as expected from the mechanism shown in Scheme 4. Of the minor products, it is striking that a significant amount of the protiated ^t^Bu_2_PCH_3_ is present as shown by the singlet at 11.5 which is ca. 20% of the intensity of the deuterated ^t^Bu_2_PCH_2_D signal. This observation could be explained by the presence of HCl in the reaction mixture, but the isotopic purity of the ethereal DCl is >95%, and therefore, it is proposed that the presence of HCl is a result of the reversible formation of deuterated ylide d_1_-**Y2** as shown in Scheme 4. This raises the possibility that protiated **Y2** (Scheme 8) may be involved in the P–Si exchange reaction, and this is further discussed below. After 72 h, complete desilylation of the P–Si reactant **1** had occurred, with the main product ^t^Bu_2_PC*H*_2_D giving an overlapping 1:1 doublet of 1:1:1 triplets (^2^ *J*_HP_ = 3.0 Hz, ^2^ *J*_HD_ = 1.5 Hz) in the ^1^H NMR spectrum and a broad featureless signal in the ^2^H NMR spectrum (see Figure S9).

**Scheme 4:**
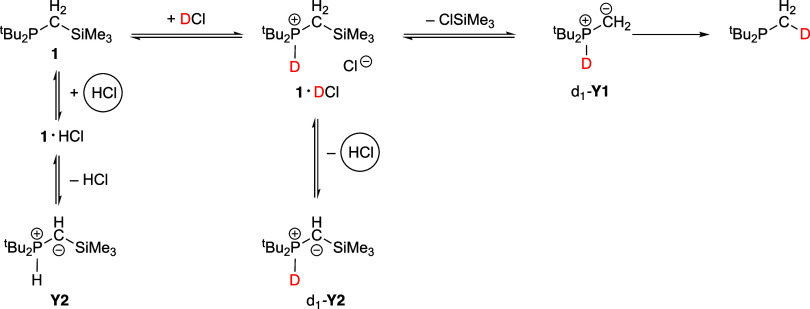
Proposed Mechanism for Deuterodesilylation of Compound **1** via Ylide Intermediate d_1_-**Y1** and Reversible Formation of d_1_-**Y2** Leading to the Presence of HCl (Circled) and Consequently Non-deuterated **Y2** ^a^

a A small peak (ca. 1%) in the ^31^P­{^1^H} NMR spectrum of the product was tentatively assigned to t-Bu_2_PCHD_2_ (see Figure S8), presumably formed via DCl addition to d_1_ *-* **Y2** followed by elimination of ClSiMe_3_.

If **1** is indeed in prototropic equilibrium with ylide **Y2**, it was postulated that **Y2** may be trapped by 2-fluorobenzaldehyde to give the intermediate **I** (Scheme 5). A Wittig-type mechanism (pathway *a* in Scheme 5) via phosphacycle **IIa** would then lead to the formation of alkene **III** and ^t^Bu_2_P­(O)­H. The reaction was carried out at ambient temperature and after 24 h, complete conversion of the aldehyde was evident from the absence of a C*H*O signal in the ^1^H NMR spectrum. However, the NMR spectra of the products were not consistent with the formation of alkene **III**. A doublet at 19.6 ppm with a small *J* of 11 Hz was present in the ^31^P­{^1^H} NMR spectrum of the product solution, indicating that ^t^Bu_2_P­(O)­H, which would have a large *J*_PH_ of 450 Hz (δ 72.4) was clearly not present.^21^ It was concluded that the doublet was due to *J*(PF) coupling and a full analysis of the ^1^H, ^13^C, ^19^F, and ^31^P NMR data led to a conclusive assignment of the product as **IV**.

**Scheme 5:**
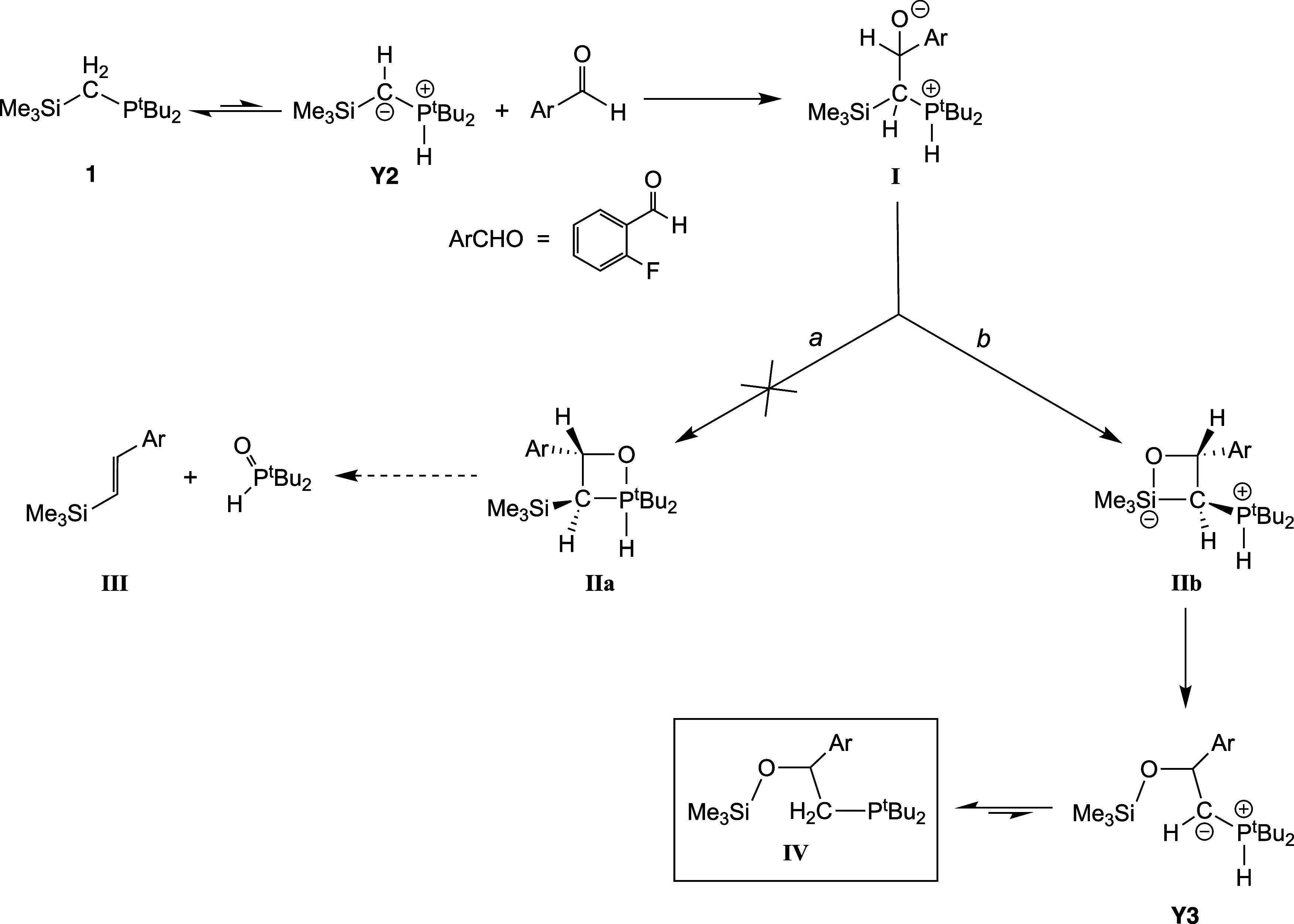
Mechanism for the Trapping of Trimethylsilyl Ylide **Y2** by 2-Fluorobenzaldehyde to Give the Siloxy Phosphine **IV** via Pathway *b* and the Formation of Another Ylide **Y3**

Pathway *b* in Scheme 5 is proposed for the formation of **IV** which involves the intermediate silacycle **IIb** and secondary phosphine ylide **Y3**. The reaction to produce **IV** can be thought of as an arrested Peterson olefination, a reaction that produces alkenes from α-silylcarbanions.^22–25^ Indeed, α-silylphosphonium ylides are known to have a preference to undergo a Peterson olefination over a Wittig reaction, due to the oxyanion having a stronger affinity for the silicon than phosphorus.^26,27^ It is notable that essentially no reaction is observed between the borane adduct ^t^Bu_2_P­(BH_3_)­CH_2_SiMe_3_ (**1**·BH_3_) and 2-fluorobenzaldehyde under the same reaction conditions,[Fn fn1] which supports the proposal of an ylide intermediate rather than an α-carbanion.

### Crossover Experiment

Further evidence for secondary phosphine ylide intermediates came from the crossover reaction that occurred between the chlorophosphite of 3,3′-diphenyl-1,1′-bi-2-naphthol (**2a**) and **3** (Scheme 6). Over a period of 16 h, the ^31^P­{^1^H} NMR spectrum of the reaction mixture evolved to show that, in addition to the signals for diphos **3** (*J*_PP_ = 117 Hz) and chlorophosphite **2a** (δ_P_ 178), there were two doublets (δ_P_, 10.5, 209.7; *J*_PP_ = 153 Hz) and a singlet at δ_P_ 180 consistent with the presence of diphos **3a** and chlorophosphite **2**.^15^ The same products in a similar ratio were obtained when the pure diphos **3a** was mixed with chlorophosphite **2**, indicating that the equilibrium shown in Scheme 6 is established over a few hours with *K* = [**3a**]­[**2]/[3**]­[**2a**] ≈ 0.08 (see the SI). This interconversion can be rationalized in terms of the secondary phosphine ylide intermediates **Y4** and **Y4a** and the common intermediate **V** shown in Scheme 7.

**Scheme 6:**
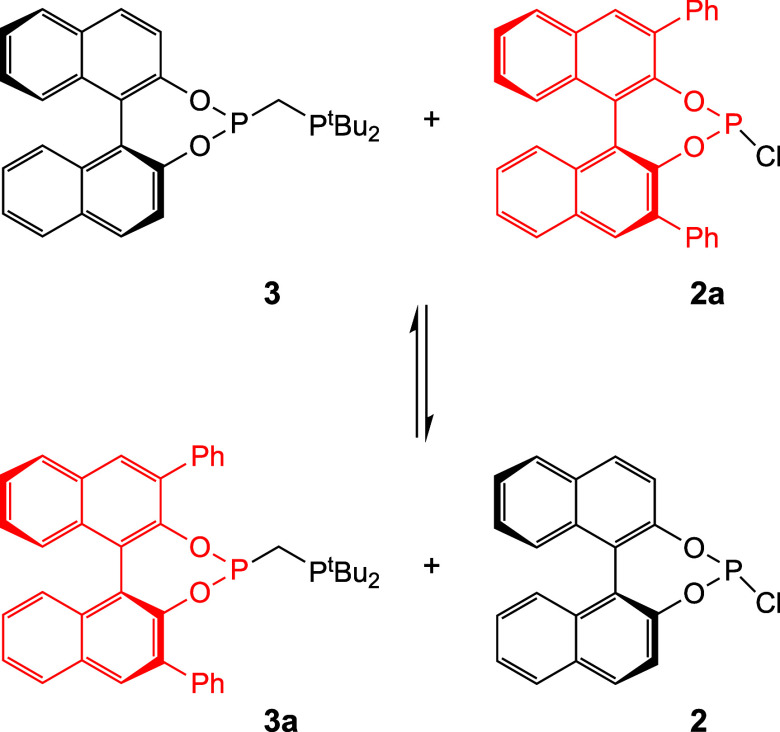
Crossover Reaction Leading to an Equilibrium Mixture of Diphos Compounds **3** and **3a**

**Scheme 7:**
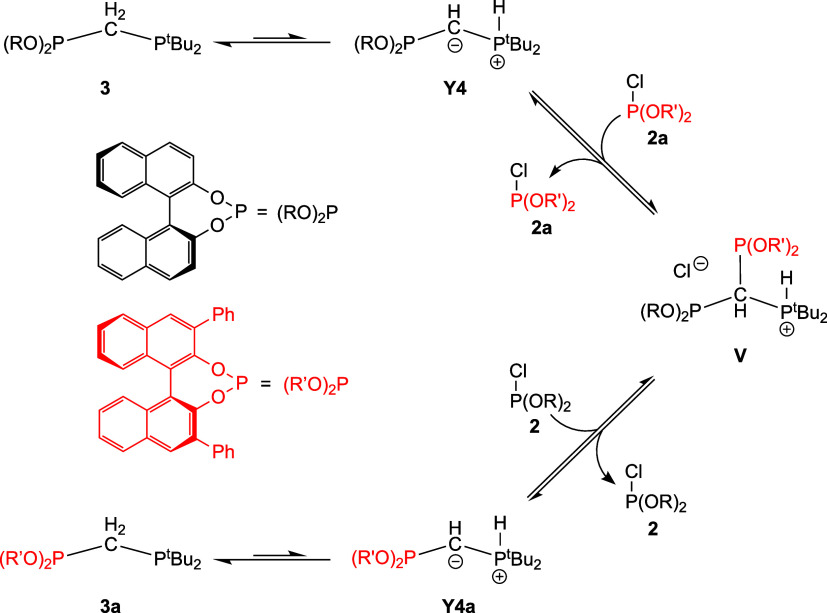
Proposed Mechanism for the Crossover Reaction Involving Secondary Phosphine Ylides **Y4** and **Y4a** and Intermediate **V**

The formation of the same ylide **Y4** can also be invoked to explain why **1**·DCl reacts with **2** to give not just the CHD compound (d_1_-**3**) but also the CD_2_ (d_2_-**3**) and CH_2_ (d_0_-**3**) isotopologues in a ratio of approximately 1:2:1 (^31^P­{^1^H} and ^1^H NMR spectra given in Figure S11). The proposed mechanism for H/D exchange via the isomeric deuterated ylides d_1_-**Y4** is shown in Scheme 8.

**Scheme 8:**
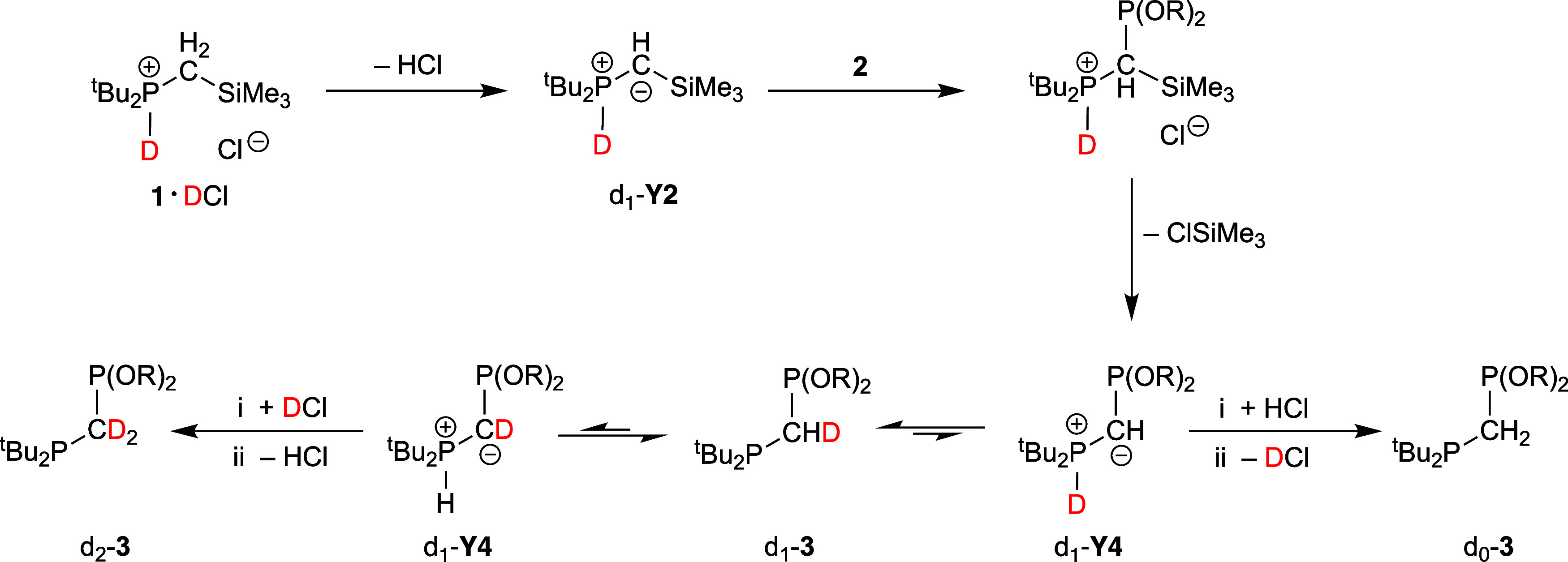
Proposed Mechanism for the Formation of d_0_, d_1_, and d_2_ Isotopologues of **3** via the Isomers of Deuterated Secondary Phosphine Ylide d_1_-**Y4** ^a^ [Fig sch7]

a The P­(OR)_2_ moiety is the P­(1,1′-bi-2-naphtholate) group identified in Scheme 7.

### Kinetic Modeling

A full mechanism model involving all chemically plausible and experimentally supported intermediates was first used for fitting. This model gave a good description of the reaction behavior in both THF and toluene over a variety of experimental conditions. However, although the involvement of the later intermediates is chemically reasonable and ylides such as **Y2** and **Y4** are supported by independent experiments, these intermediates are not directly observed during the in situ monitoring because of low concentrations. At the same time, these later steps are faster than formation of the ylide **Y2**, which is the turnover-limiting step (TLS) of the reaction and therefore have less influence on the overall temporal concentration profiles. Forcing a global fit with multiple, poorly constrained late-stage parameters would therefore risk overfitting. To obtain a more robust and chemically meaningful description of the system, a simplified model (unshaded region in Scheme 9) was used for quantitative analysis.

**Scheme 9:**
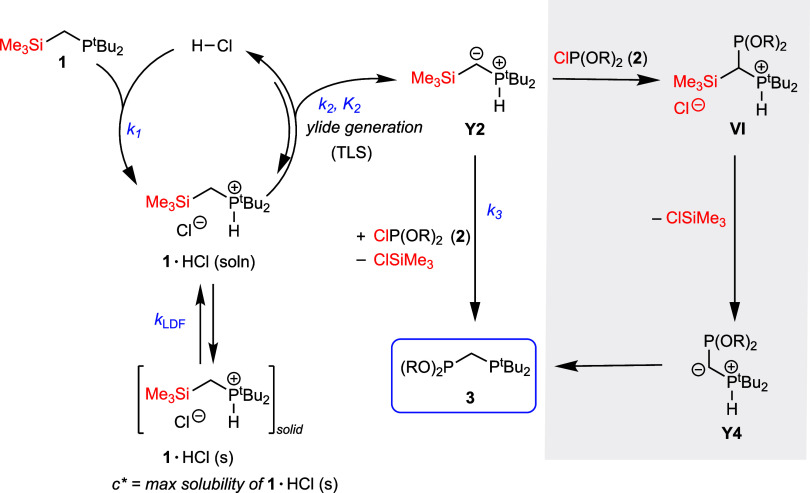
Proposed Mechanism for Kinetic Simulation and Fitting Purposes^a^

a The rate and equilibrium constants used are shown in Table 2. The late-stage species in the gray box were not included in the kinetic model (see Results and Discussion section).

The kinetics have been simulated according to the model for the forward reaction shown in Scheme 9 for the two solvents toluene and THF using the temporal concentration data previously obtained. For reactions in THF, the simplified treatment additionally included precipitation/dissolution of **1**·HCl. This was implemented using a linear driving force mass-transfer term with an effectively large mass-transfer coefficient, *k*_LDF_, so that the dissolved concentration of **[1**·HCl**]** does not exceed its maximum solubility (for more details, see Scheme S1).

From the simulation, the rate constants (*k*_1_–*k*_3_) and equilibrium constants (*K*_2_) were derived and are given in Table 2. The large value for the rate constant *k*_1_ in toluene (1.09 × 10^2^ M^–1^ s^–1^) and THF (3.66 × 10^1^ M^–1^ s^–1^) reflects the rapid protonation of **1** to give **1**·HCl. The small value for *k*_2_ in toluene (2.87 × 10^–1^ s^–1^) and THF (5.35 × 10^–1^ s^–1^) reflects the slow ylide **Y2** formation in the turnover-limiting step. Similarly, the low value of the equilibrium constant *K*_2_ (6.87 × 10^–6^ M in toluene and 8.83 × 10^–5^ M in THF) for the turnover-limiting-step reflects the equilibrium being largely in favor of the phosphonium salt **1**·HCl as opposed to the ylide **Y2**, which is as expected, and in agreement with experimental observations.

**Table 2:** Rate and Equilibrium Constants for Model Used to Simulate the Mechanism Proposed in Scheme 9 ^a^

entry	*k* or *K*	toluene	THF
1	*k*_1_	1.09 × 10^2^ M^–1^ s^–1^	3.66 × 10^1^ M^–1^ s^–1^
2	*k*_2_	2.87 × 10^–1^ s^–1^	5.35 × 10^–1^ s^–1^
3	*K*_2_	6.87 × 10^–6^ M	8.83 × 10^–5^ M
4	*k*_3_	2.72 M^–1^ s^–1^	1.04 × 10^2^ M^–1^ s^–1^
5	*k*_LDF_	N/A	≥1 × 10^3^ s^–1^
6	c*	N/A	1.9 × 10^–4^ M

a See the SI for full details of values and errors.

After the turnover-limiting-step, the undetected ylide **Y2** is proposed to react with the halophosphite to form the diphos intermediate **VI**. Subsequent elimination of ClSiMe_3_ leads to the formation of the diphos ylide **Y4**, which rearranges to form the isomeric diphos product. While these intermediates are not involved in the simplified model, the irreversible last step to product **3** with large value for *k*_3_ (2.72 in toluene and 1.04 × 10^2^ in THF) reflects the intermediates lying toward the non-ylide diphos product, which is consistent with experimental observations.

The results of the kinetic simulations give satisfactory correlations between the experimental and simulated data, supporting the proposed mechanism (Scheme 9). Specifically, as shown by the plots in Figure S7 (see the SI), the simulations produced from the one model with one set of rate and equilibrium constants fit across a wide range of experimental data, involving several initial concentrations of reagents (**1** and **2**) and of HCl (see Figure 5).

**Figure 5:**
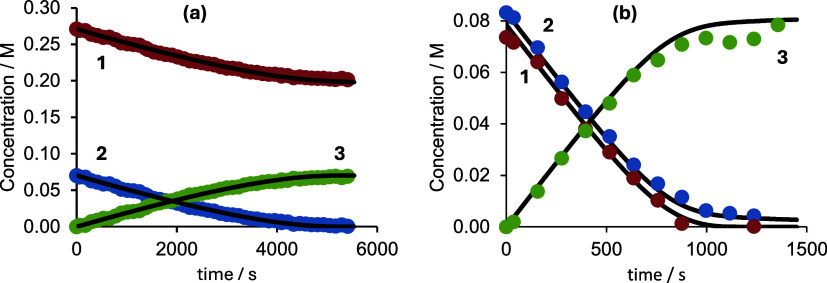
Examples of temporal concentration data for the reaction of **1** with **2**. The circles correspond to experimentally determined data from ^31^P­{^1^H} NMR spectra (red = **1**, blue = **2**, green = **3**), and the lines correspond to simulated data according to Scheme 9 using rate and equilibrium constants shown in Table 2. Initial conditions: (a) [**1**]_0_ = 0.27 M, [**2**]_0_ = 0.08 M in toluene at 40 °C. (b) [**1**]_0_ = [**2**]_0_ = 0.08 M in THF with 5% HCl (1 M in Et_2_O) added; note that the reaction is significantly faster in THF in the presence of HCl and displacement of the curves for **1** and **2** is due to the precipitation of **1**·HCl. See the SI for more examples of data plots. In all instances, *R* ^2^ values for the fit of simulated to experimental data are >0.98.

## Conclusions

The observations that have led to the proposed mechanism in Scheme 9 are summarized as follows. (1) No reaction was observed when using the borane adduct of the phosphino­silane, ^t^Bu_2_P­(BH_3_)­CH_2_SiMe_3_ (**1**·BH_3_). (2) The reaction with respect to both **1** and **2** was found to be (pseudo) zero order. (3) A first-order dependence was observed between the *initial* concentration of chlorophosphite, but extrapolation of the trend line produced a non-zero intercept, suggesting that there was an impurity present in the bulk of the chlorophosphite responsible for catalyzing the reaction. (4) HCl was identified as an adventitious catalyst, but kinetic saturation behavior was observed at higher concentrations which can be explained by the insolubility of the protonated [^t^Bu_2_P­(H)­CH_2_SiMe_3_]Cl (**1**·HCl) which effectively buffered the [HCl]. (5) The reaction between **1** and **2** was completely inhibited by the presence of the base NEt_3_. (6) The SiMe_3_ group is important in allowing the forward reaction to proceed via loss of ClSiMe_3_. No reaction was observed between ^t^Bu_2_PMe and chlorophosphite **2**. (7) Evidence for an ylide intermediate and the importance of the SiMe_3_ group was provided by the Peterson-like reaction upon addition of an aldehyde to the phosphinosilane reactant **1**. (8) A crossover experiment provided further evidence for an ylide intermediate; addition of the chlorophosphite derived from 3,3′-diphenyl-bi-2-naphtholate (**2a**) to the diphos **3** led to the formation of **3a** and the unsubstituted chlorophosphite **2**, and this process was shown to be reversible. (9) Powerful support for the proposed mechanism comes from the kinetic simulations of the model which provide an excellent fit with the experimental data.

The overall conclusion is that, while stable secondary phosphine ylides are rare and involve highly electron-withdrawing substituents on the C,^1,10–14^ the evidence presented here supports the involvement of secondary phosphine ylides of the type ^t^Bu_2_P­(H)=CHZ, where Z = H (**Y1**), SiMe_3_ (**Y2**), alkyl (**Y3**), or P­(OR)_2_ (**Y4**). While none of these Z substituents is particularly electronegative, in the case of **Y2** and **Y4**, the negative charge (evident in the dipolar canonical form of the ylides shown in Scheme 10) can be delocalized via negative hyperconjugation with the third-row element substituent.^28,29^ The results reported herein raise the possibility that secondary phosphine ylide intermediates of the type R_2_P­(H)=CHZ may be involved in the mechanisms of other organophosphorus reactions.

**Scheme 10:**
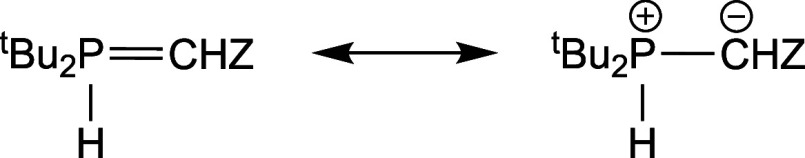
Canonical Forms of the Secondary Phosphine Ylides

## Experimental Section

No uncommon hazards were noted. Unless otherwise stated, all reactions were carried out under an argon atmosphere using standard Schlenk-line techniques. Dry N_2_-saturated solvents were collected from a Grubbs system^30^ in flame- and vacuum-dried glassware. All phosphines were stored under a nitrogen atmosphere at room temperature. All other reagents were used as received from Aldrich, Strem, or Lancaster. NMR spectra were recorded on a Jeol ECP300, Jeol Eclipse 400, or Varian 400 spectrometer. Mass spectra were recorded on an MD800 spectrometer by the Mass Spectrometry Service, University of Bristol.

### General Procedure for the Kinetic Measurements of the Reaction between ^t^Bu_2_PCH_2_SiMe_3_ (1) and PCl­(1,1′-bi-2-naphtholate) (2) to Give ^t^Bu_2_PCH_2_P­(1,1′-bi-2-naphtholate) (3) in Toluene or THF

In an argon atmosphere glovebox, **1** and **2** were weighed into separate oven-dried vials. Both compounds were then separately dissolved in dry, Ar-saturated toluene (1.00 mL in total) and then the solutions mixed. The resultant reaction mixture was then transferred by syringe to a J-Young NMR tube which was then removed from the box before being cooled to 0 °C. The NMR tube was then placed into a preheated (40 °C) spectrometer and timing started (*t* = 0) for the reaction monitoring by ^31^P­{^1^H} NMR spectroscopy. The spectra were all collected on a Jeol ECP300 spectrometer operated in a way to make the integration of the signals reliably represent the ratio of the concentrations of **1**, **2**, and **3**: inverse gated decoupling was used to minimize the Nuclear Overhauser Effect (NOE), a relaxation delay of 7 s was used to minimize saturation effects, and 8 scans were accumulated for each data point in the experiment. Typical concentration vs time plots that were obtained are shown in the SI (Figures S1–S4) with initial conditions for each experiment given in the figure captions. When the reactions were carried out under pseudo-first-order conditions, one of the reagents was in considerable excess. Details of the individual experiments including the parameters used in the kinetic modeling are given in the SI.

### NMR Study of the Chlorodesilylation of ^t^Bu_2_PCH_2_SiMe_3_ (1) with DCl

Under Ar, a solution of **1** (10 mg, 0.040 mmol) in THF (0.50 mL) was treated with a THF/Et_2_O solution of DCl (0.50 mL, 0.080 M, 0.040 mmol), and the mixture was shaken and then left to stand. The ^31^P­{^1^H} NMR spectrum, recorded after 24 and 72 h, showed that there was no remaining **1** after 72 h and that 2 major products (^t^Bu_2_PCH_2_D and ^t^Bu_2_PCH_3_) were formed along with a small amount (ca. 1%) of a minor product tentatively assigned to ^t^Bu_2_PCHD_2_ based on its δ_P_ value and confirmed by ^1^H NMR spectroscopy (see Figures S8 and S9 in the SI).

### Preparation of ^t^Bu_2_PCH_2_CHAr­(OSiMe_3_) (IV, Ar = *o-*C_6_H_4_F) via a Peterson-like Reaction

A solution of **1** (25 mg, 0.10 mmol) in THF (1 mL) was added to a solution of 2-fluorobenzaldehyde (14 mg, 0.11 mmol) in THF (1 mL) and the mixture stirred at ambient temperature for 16 h. The volatiles were then removed under reduced pressure to give **IV** as a colorless oil which was characterized by NMR spectroscopy only. ^31^P­{^1^H} NMR (122 MHz, CD_2_Cl_2_): d 19.60 (d, ^5^ *J*_PF_ = 11.2 Hz). ^19^F­{^1^H} (282 MHz, CD_2_Cl_2_): d – 119.60 (d, ^5^ *J*_PF_ = 11.2 Hz). ^1^H NMR (300 MHz, CD_2_Cl_2_): d 0.05 (9H, s, SiMe_3_); 1.05 (9H, d, ^3^ *J*_PH_ = 10.7 Hz, CMe_3_); 1.13 (9H, d, ^3^ *J*_PH_ = 10.9 Hz, CMe_3_); 1.84 (2H, m, CH_2_); 5.09 (1H, apparent q, *J* = 6.7 Hz, CHO); 6.99 (1H, m, ArH); 7.10–7.27 (2H, m, ArH); 7.51 (1H, m, ArH).

### Crossover Experiments

A solution of **3** (10 mg, 0.021 mmol) in THF (0.5 mL) was added to a solution of PCl­(3,3′-diphenyl-2,2′-binaphthyldiolate), **2a** (11 mg, 0.022 mmol) in THF (0.5 mL) in a Young’s NMR tube, and the mixture was shaken vigorously for 2 min; no immediate reaction was apparent by ^31^P NMR spectroscopy after 10 min, but after 16 h, the ^31^P NMR spectrum shown in Figure S10 (see the SI) was obtained. This spectrum is consistent with a mixture of **3** and ^t^Bu_2_PCH_2_P­(3,3′-diphenyl-2,2′-binaphthyldiolate) (**3a**) as well as chlorophosphites **2** and **2a** in the ratios indicated in Figure S10. A very similar spectrum was obtained 16 h after a solution of **3a** (13 mg, 0.021 mmol) in THF (0.5 mL) was added to a solution of **2** (7 mg, 0.020 mmol) in THF (0.5 mL) in a Young’s NMR tube, and the mixture was shaken vigorously for 2 min. This is consistent with the measured proportions of **3**/**3a** and **2**/**2a** corresponding to an equilibrium mixture.

## Supplementary Material



## Footnotes

1 Some product IV was formed (approximately 5% after 72 h) upon refluxing **1**·BH_3_ with 2-fluorobenzaldehyde in toluene which is likely derived from borane dissociation from **1**·BH_3_.

## Notes

Details of experimental method for the ^31^P­{^1^H} NMR kinetic studies of the reaction of **1** with **2**; kinetic modeling of the results obtained under a range of conditions and plots showing the fit with experimental data; and ^1^H, ^2^H, and ^31^P­{^1^H} NMR spectra for reaction of **1** with DCl and ^31^P­{^1^H} NMR spectra for the crossover experiments shown in Scheme 7 (PDF)
