# Supplementary material for: Evidence for Transient Ylide Intermediates of the Type tBu2P(H)=CHZ That Are Prototropic Tautomers of tBu2PCH2Z
Source: Inorg Chem. 2026 Jun 29;65(27):15866–75. doi: 10.1021/acs.inorgchem.6c02065 (PMC13370874; doi:10.1021/acs.inorgchem.6c02065)
Supplement: Supplementary file 1 [file ic6c02065_si_001.pdf]

Supporting Information

Evidence for transient ylide intermediates of the type $^tBu_2P(H)=CHZ$ that are Prototropic Tautomers of $^tBu_2PCH_2Z$

E. Louise Hazeland,[a] Chen Li,[b] Nicholas P. Taylor,[b] Guy C. Lloyd-Jones,[b]* and Paul G. Pringle[a]*

[a] *School of Chemistry, University of Bristol, Cantocks Close, Bristol, BS8 1TS, UK*

[b] *School of Chemistry, University of Edinburgh, Joseph Black Building, David Brewster Road, Edinburgh, EH9 3FJ, UK*

Email: guy.lloyd-jones@ed.ac.uk or paul.pringle@bristol.ac.uk

Contents **Page**

S1 General Procedure for the kinetic measurements of the reaction between ${}^{t}Bu_2PCH_2SiMe_3$ (**1**) and PCl(1,1'-bi-2-naphtholate) (**2**) to give ${}^{t}Bu_2PCH_2P$(1,1'-bi-2-naphtholate) (**3**) in toluene or THF S3

S2 General Procedure for the kinetic measurements of the reaction between ${}^{t}Bu_2PCH_2SiMe_3$ (**1**) and PCl(1,1'-bi-2-naphtholate) (**2**) to give ${}^{t}Bu_2PCH_2P$(1,1'-bi-2-naphtholate) (**3**) in THF in the presence of HCl or other additives S8

S3 Kinetic Modelling S8

S4 NMR study of the chlorodesilylation of ${}^{t}Bu_2PCH_2SiMe_3$ with DCl S17

S5 Preparation of ${}^{t}Bu_2PCH_2CHAr(OSiMe_3)$ Ar = *o*-C_6H_4F) via a Peterson-like reaction S18

S6 Crossover experiments S18

S7 NMR study of the reaction between $[{}^{t}Bu_2P(D)CH_2SiMe_3]Cl$ (**1**·DCl) and **2** S19

No uncommon hazards are noted. Unless otherwise stated, all reactions were carried out under argon using standard Schlenk-line techniques. Dry N_2-saturated solvents were collected from a Grubbs system[1] in flame and vacuum-dried glassware. All phosphines were stored under nitrogen at room temperature. Phosphines **1**-**3** were made using previously described procedures and all other reagents[2] were used as received from Aldrich, Strem or Lancaster. NMR spectra were recorded on a Jeol ECP300, Jeol Eclipse 400 or Varian 400. Mass spectra were recorded on a MD800 by the Mass Spectrometry Service, University of Bristol.

S1 General Procedure for the kinetic measurements of the reaction between $^tBu_2PCH_2SiMe_3$ (1) and PCl(1,1'-bi-2-naphtholate) (2) to give $^tBu_2PCH_2P$(1,1'-bi-2-naphtholate) (3) in toluene or THF

In an argon atmosphere glove box, **1** and **2** were weighed into separate oven-dried vials. Both compounds were then separately dissolved in dry, argon-saturated toluene (1.00 mL in total) and then the solutions mixed. The resultant reaction mixture was then transferred by syringe to a J-Young NMR tube which was then removed from the box before being cooled to 0 ˚C. The NMR tube was then placed into a preheated (40 ˚C) spectrometer and timing started (t = 0) for the reaction monitoring by $^{31}P\{^1H\}$ NMR spectroscopy. The spectra were all collected on a Jeol ECP300 operated in a way to make the integration of the signals reliably represent the ratio of the concentrations of **1**, **2** and **3**: inverse gated decoupling was used to minimise the Nuclear Overhauser Effect (NOE), a relaxation delay of 7 s was used to minimise saturation effects, and 8 scans were accumulated for each data point in the experiment. A typical stacked plot from which the integrals for **1**, **2** and **3** were measured is shown below (where **2** was used in excess):

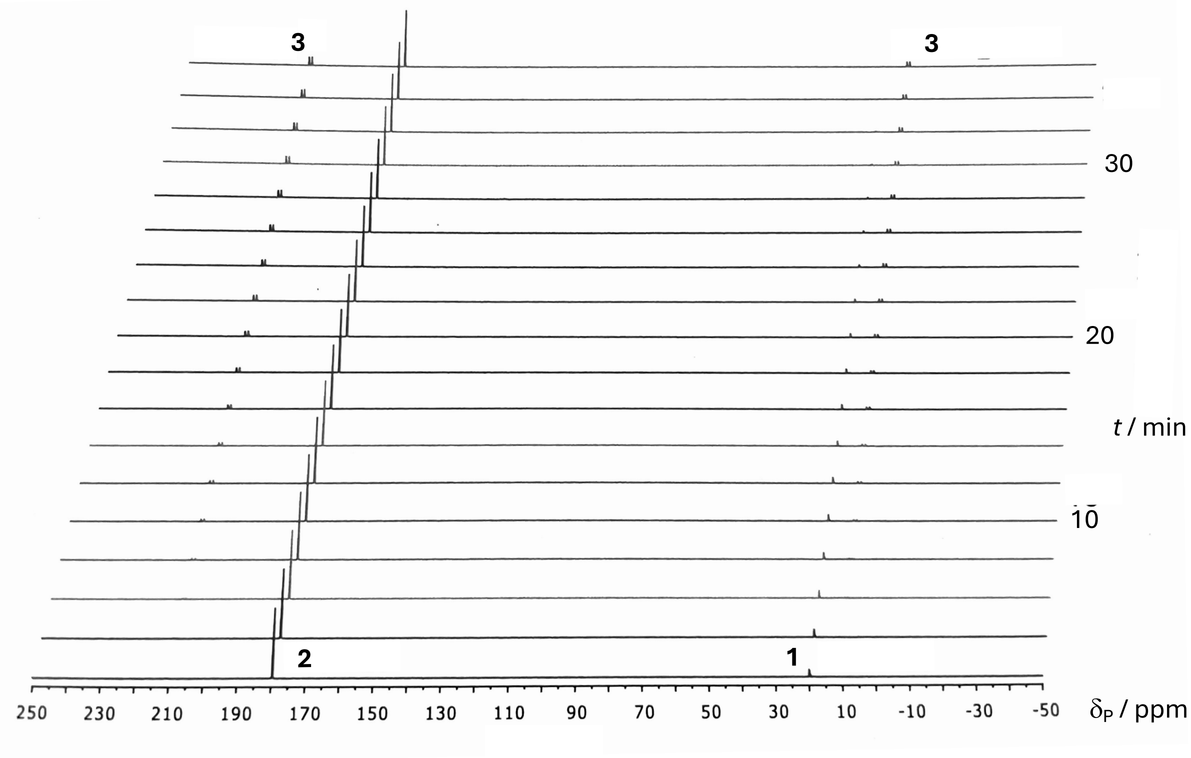

Typical concentration vs time plots that were obtained are shown in Figures S1–S4 with initial conditions for each experiment given in the figure captions. Figures S1 and S2 are under the same conditions but in toluene and THF respectively and a comparison of the two Figures shows that the reaction occurs more rapidly in THF than toluene. The reaction was carried out under pseudo first order conditions in which one of the reagents is in considerable excess. Thus Figure S3(a) shows the reaction carried out in the presence of an excess of **2** and the non-linearity of log plot shown in Figure S3(b) indicates that the reaction is not first order in [**1**]. Similarly, Figure S4(a) shows the reaction carried out in the presence of an excess of **1** and the non-linearity of log plot shown in Figure S4(b) indicates that the reaction is not first order in [**2**].

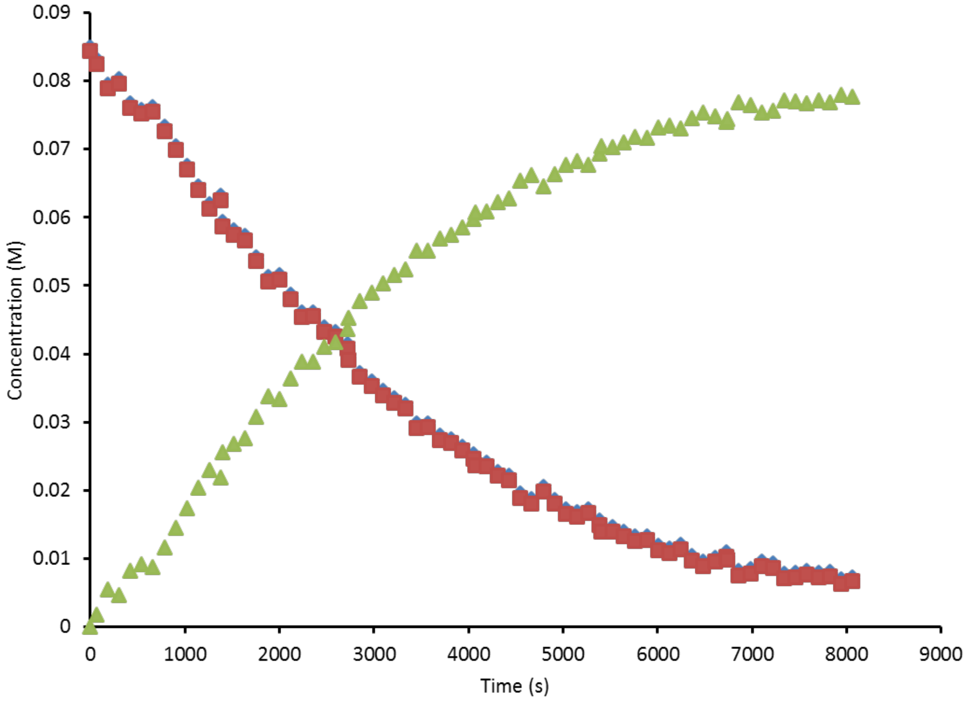

Figure S1: Profiles for the reaction of **1** (red squares) with **2** (blue diamonds) to give **3** (green triangles). Initial concentrations: $[\mathbf{1}]_0 = [\mathbf{2}]_0 = 0.084$ M in toluene at 40 ˚C. The datapoints for **2** are obscured by the datapoints for **1** because they have essentially the same values.

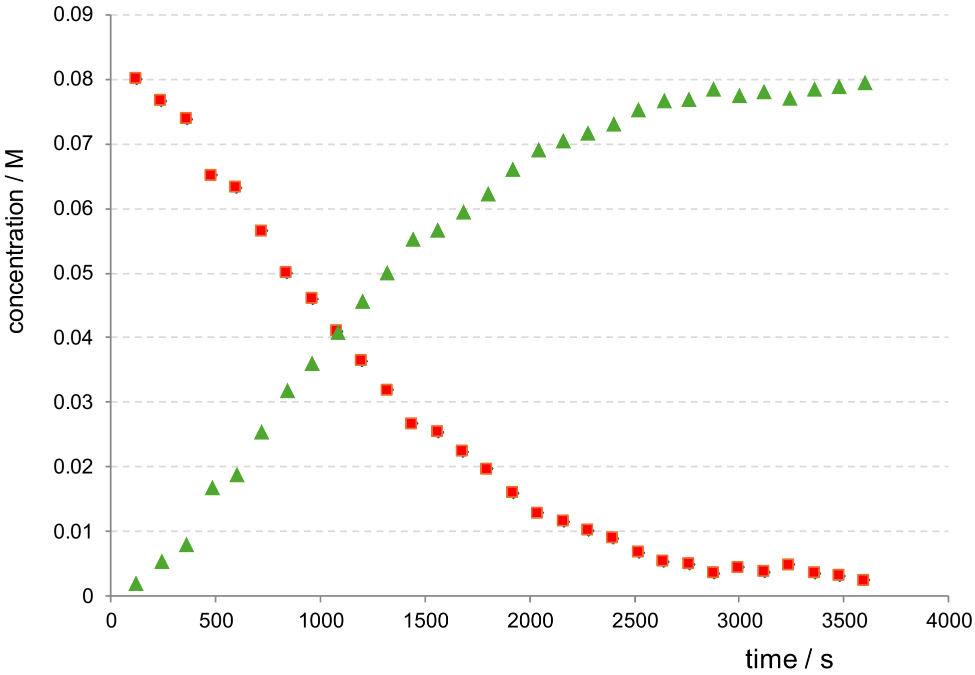

Figure S2: Profiles for the reaction of **1** (red squares) with **2** (blue diamonds) to give **3** (green triangles). Initial concentrations: $[\mathbf{1}]_0 = [\mathbf{2}]_0 = 0.084$ M in THF at 40 ˚C. The datapoints for **2** are obscured by the datapoints for **1** because they have essentially the same values. This plot should be compared with Figure S1 which is under similar conditions; the higher rate of reaction in THF is clear from the time taken to 50% completion: *ca.* 1100 s in THF, *ca.* 2800 s in toluene.

(a)

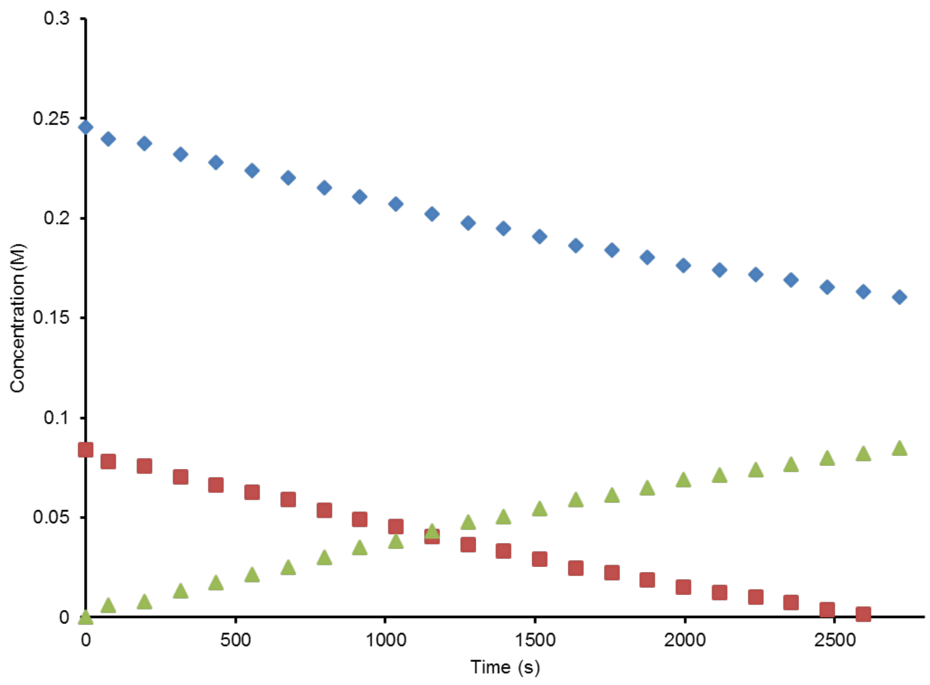

(b)

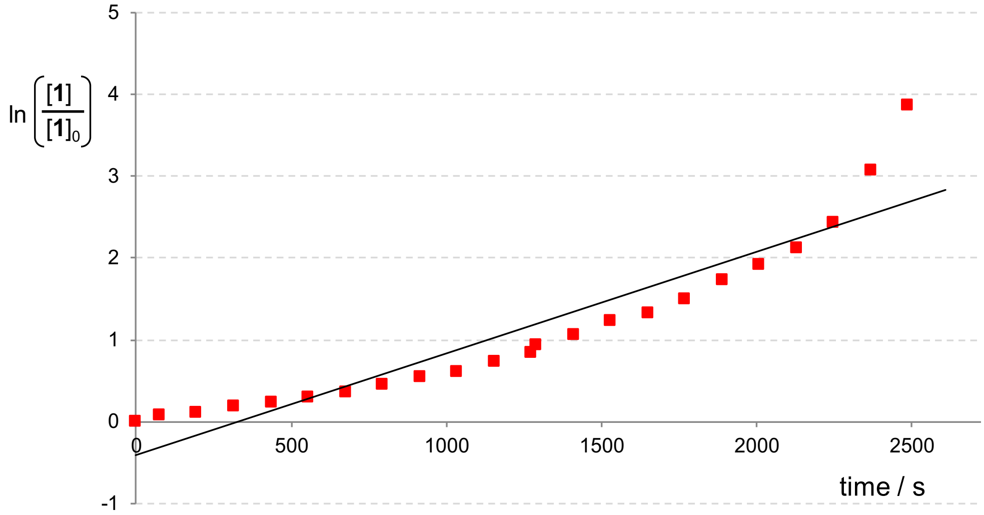

Figure S3: (a) Profiles for the reaction of **1** (red squares) with **2** (blue diamonds) to give **3** (green triangles). Initial concentrations: [**1**]$_0$ = 0.084 M, [**2**]$_0$ = 0.250 M, in toluene at 40 ˚C. (b) Plot of ln{[**1**]/[**1**]$_0$} against time for the data in the upper plot (a) showing a poor linear fit and therefore inconsistent with the rate law being first order in [**1**].

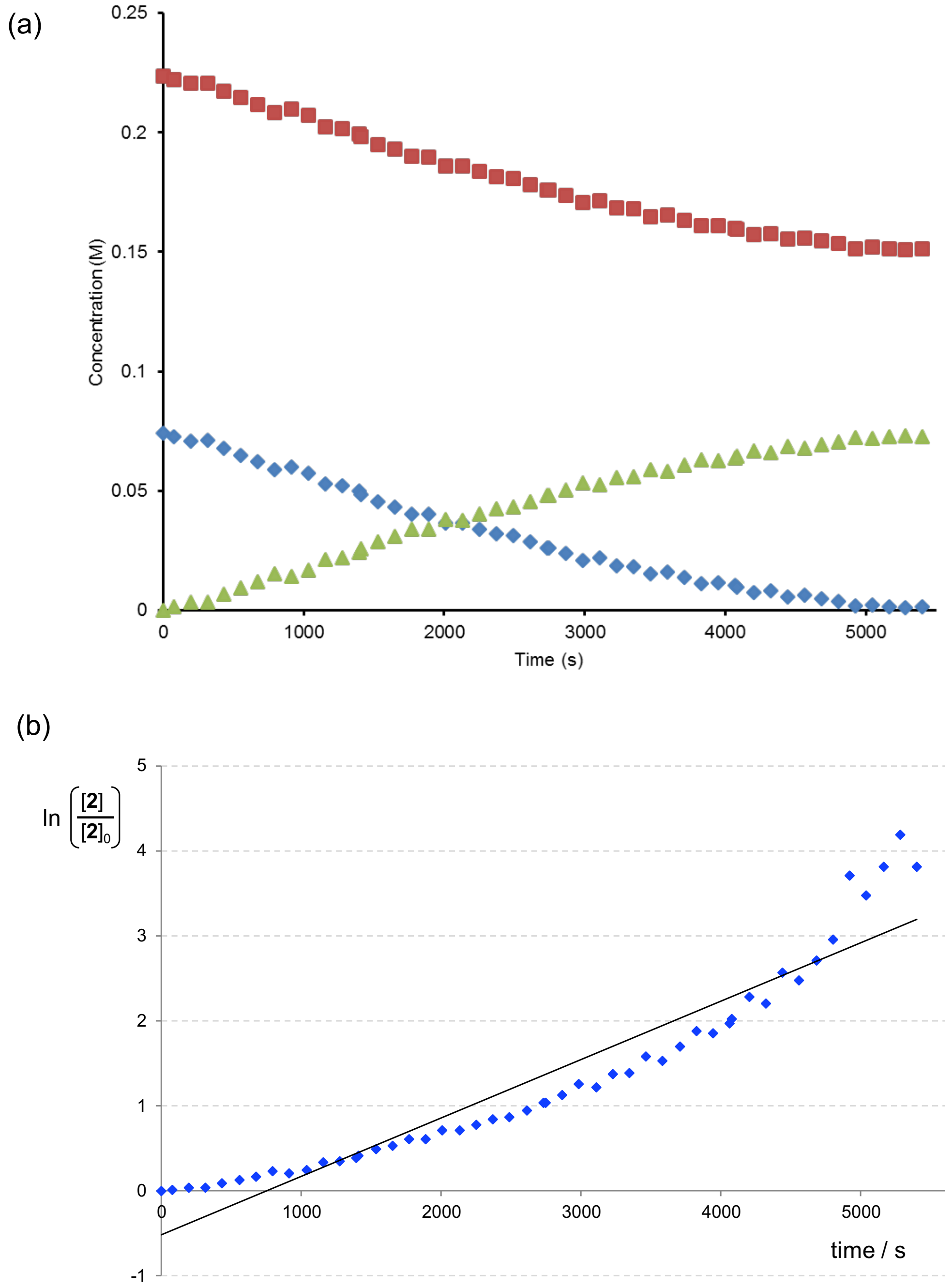

Figure S4: (a) Profiles for the reaction of **1** (red squares) with **2** (blue diamonds) to give **3** (green triangles). Initial concentrations: $[\mathbf{1}]_0 = 0.220$ M, $[\mathbf{2}]_0 = 0.075$ M, in toluene at 40 ˚C. (b) Plot of $\ln\{[\mathbf{2}]/[\mathbf{2}]_0\}$ against time for the data in the upper plot (a) showing a poor linear fit and therefore inconsistent with the rate law being first order in [**2**].

S2 General Procedure for the kinetic measurements of the reaction between $^tBu_2PCH_2SiMe_3$ (1) and PCl(1,1'-bi-2-naphtholate) (2) to give $^tBu_2PCH_2P$(1,1'-bi-2-naphtholate) (3) in THF in the presence of HCl or other additives.

In an argon atmosphere glove box, **1** and **2** were weighed into separate oven-dried vials. Both compounds were then separately dissolved in dry, argon-saturated THF (0.50 mL in total) and then the solutions mixed. The resultant reaction mixture was then transferred by syringe to a screw cap NMR tube which was then removed from the box before being cooled to 0 ˚C. Under argon, a stock solution of HCl (0.50 mL, varying in concentration from 6.4×10^{-3} M to 4.0×10^{-5} M) was then added by syringe to the reaction mixture and the NMR tube inverted to mix the solutions. The NMR tube was then placed into a preheated (40 ˚C) spectrometer for the reaction monitoring by $^{31}P\{^1H\}$ NMR spectroscopy, as described in procedure S1 above. For solubility reasons, testing the effects of other additives (see Table 1 of the main article) were also carried out in THF.

S3 Kinetic Modelling

As shown in Figure 4 in the manuscript, the initial protonation step, **1** + HCl → **1·HCl**, is rapid and essentially complete before the first experimental data point is acquired, it has only a limited influence on the subsequent temporal concentration profiles. However, to enable a more reliable fit for its rate constant, k_1, and the maximum solubility of **1·HCl**, the initial concentrations for kinetic simulations were set as the theoretical concentrations (rather than the first experimental data) immediately after mixing. In addition to these considerations, the inherent uncertainty in the concentration of HCl generated in situ from the hydrolysis of **2** by the adventitious water present, and the precipitation of [**1·HCl**] means that the values of rate and equilibrium constants should not be used in isolation. To obtain the values in Scheme S1, an estimation of the value of $[HCl]_0$ was required which was achieved using the calibration curve shown in Figure S5. Full experimental details are given in Tables S1 and S2 for experiments conducted in THF and toluene, respectively.

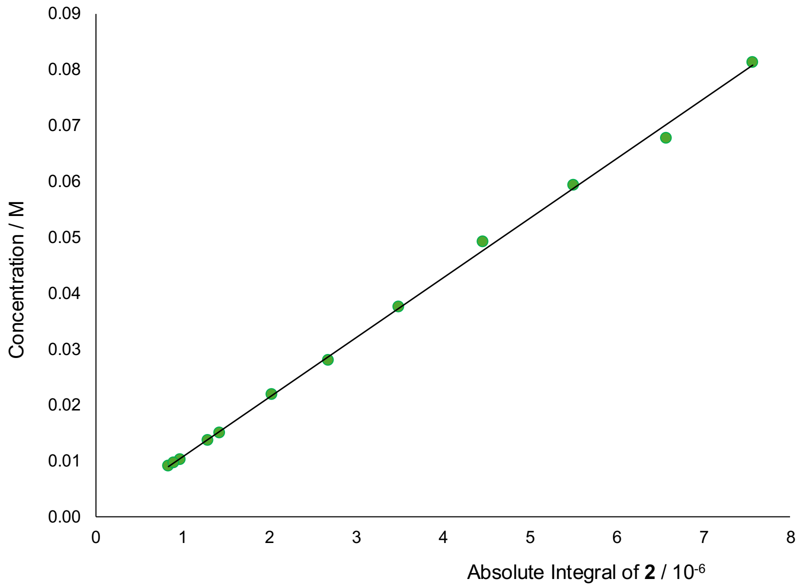

Figure S5: Calibration curve. From the absolute integral of the ^{31}P NMR signal for **2**, in THF from which $[HCl]_0$ can be calculated.

Table S1. Experimental conditions for in situ monitoring in THF at different initial HCl concentrations.

Entry	initial $[1]_0$ / M [a]	initial $[2]_0$ / M [a]	initial $[HCl]_0$ / M [a]	Added [HCl] / M
S1(1)	8.43E-02	8.30E-02	1.37E-02	1.36E-02
S1(2)	8.22E-02	8.33E-02	6.67E-03	6.57E-03
S1(3)	8.22E-02	8.30E-02	5.85E-03	5.75E-03
S1(4)	8.26E-02	8.33E-02	4.22E-03	4.13E-03
S1(5)	8.35E-02	8.38E-02	3.43E-03	3.34E-03
S1(6)	8.35E-02	8.33E-02	2.60E-03	2.50E-03
S1(7)	8.35E-02	8.38E-02	1.76E-03	1.67E-03
S1(8)	8.35E-02	8.30E-02	5.08E-04	4.15E-04
S1(9)	8.26E-02	8.30E-02	3.33E-04	2.40E-04
S1(10)	8.39E-02	8.30E-02	1.33E-04	4.00E-05
S1(11)	8.22E-02	8.27E-02	9.27E-05 [b]	0.00E+00

[a] Theoretical initial concentrations after mixing, prior to the start of the reaction. Initial $[HCl]_0$ was calculated as the sum of added HCl and HCl formed in situ from residual water in the solvent (represented by entry S1(11)).

[b] Estimated trace HCl generated by hydrolysis of ClP(1,1′-bi-2-naphtholate) (**2**) by residual water in the solvent.

Table S2. Experimental conditions for in situ monitoring in toluene with varied initial [**1**]:[**2**] ratio.

Entry	initial $[1]_0$ / M [a]	initial $[2]_0$ / M [a]	initial $[HCl]_0$ / M [a, b]	Initial $[1]_0$: $[2]_0$
S2(1)	2.24E-01	7.41E-02	8.41E-05	3.02
S2(2)	8.43E-02	8.50E-02	9.50E-05	0.99
S2(3)	8.99E-02	1.78E-01	1.88E-04	0.50
S2(4)	8.39E-02	2.45E-01	2.55E-04	0.34
S2(5)	8.52E-02	3.20E-01	3.30E-04	0.27
S2(6)	2.71E-01	7.01E-02	8.01E-05	3.86
S2(7)	1.39E-01	6.96E-02	7.96E-05	2.00

[a] Theoretical initial concentrations after mixing, prior to the start of the reaction.

[b] Estimated trace HCl generated by hydrolysis of ClP(1,1'-bi-2-naphtholate) (**2**) by residual water in the solvent.

Initially, a full mechanism model involving all evidenced species (as shown in Scheme 9 in the main manuscript) was used for fitting. For an indication of the global fit of the model, a minimum R^2 value is given in all the plots shown in Figures S6.

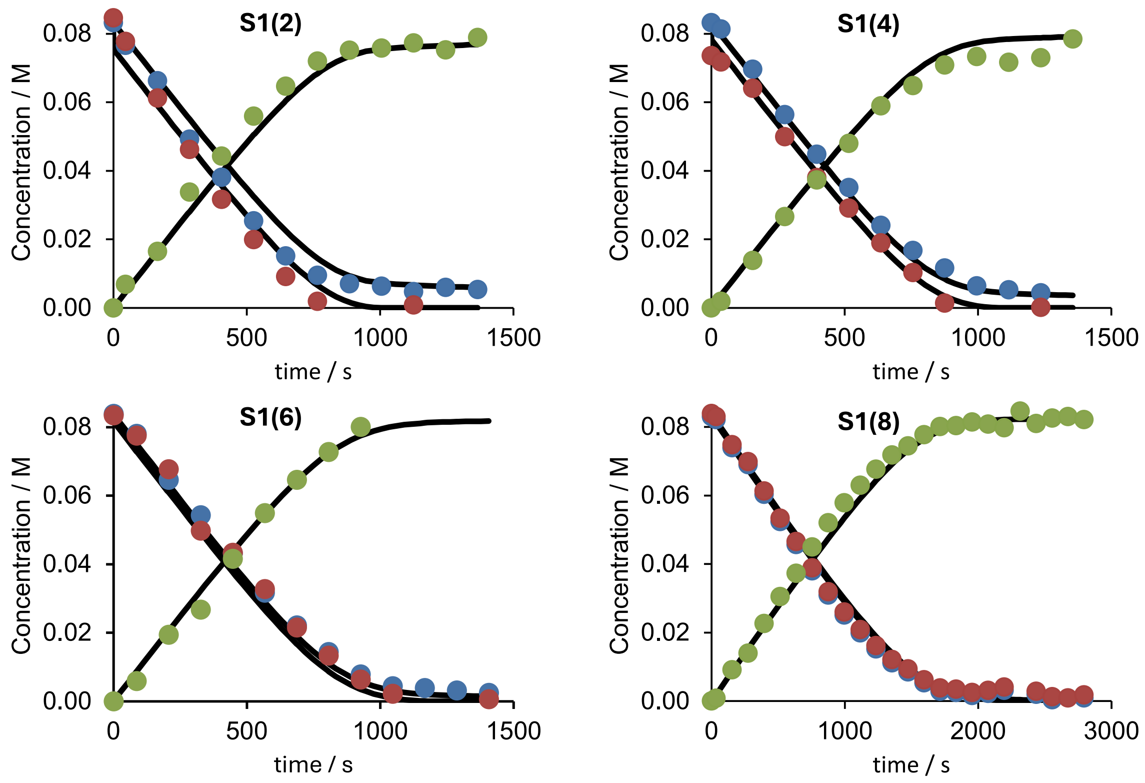

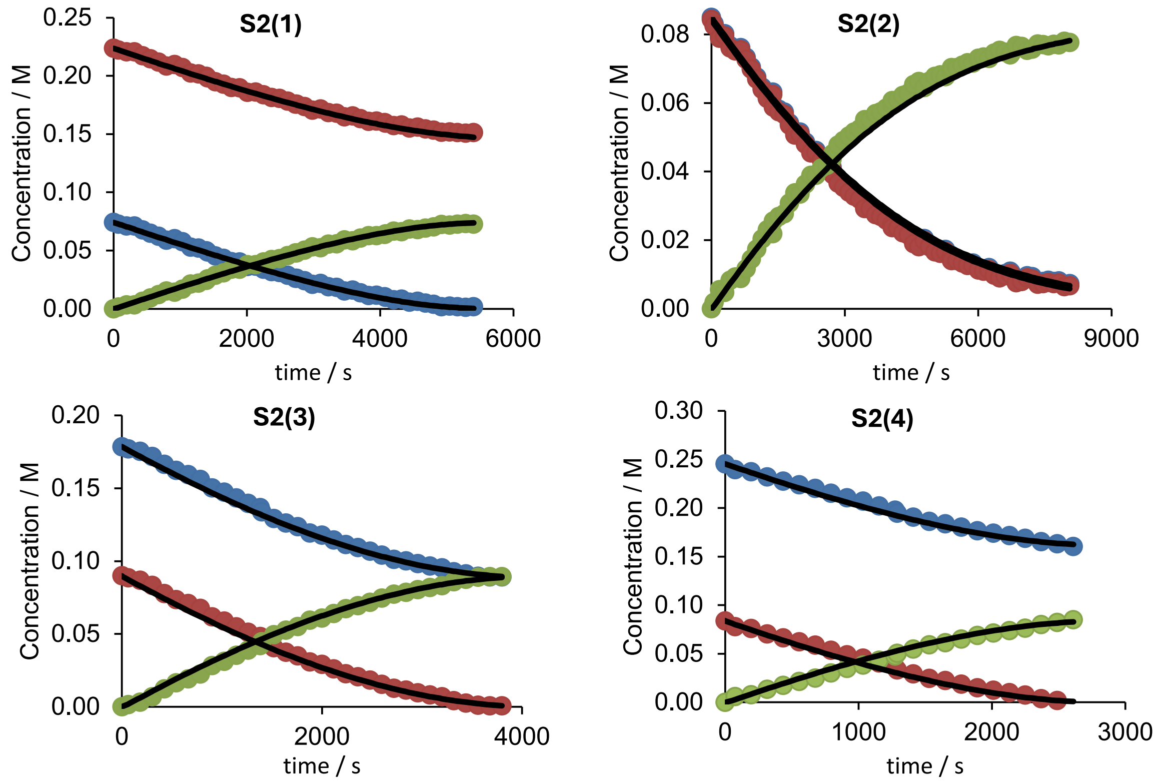

Figure S6: Selected reaction profiles for the conversion of **1** (red) and **2** (blue) to **3** (green), simulated with the full model (Scheme 9, main manuscript). Initial concentrations were taken from Tables S1 (entries S1(1)–S1(8) in THF) and **S2** (entries S2(1)–S2(4) in toluene) at 40 °C. Circles represent the experimental data obtained by integration of the $^{31}P\{^1H\}$ NMR spectra, and solid lines represent the simulated data. The R^2 values for all three profiles are > 0.98.

As shown by the fitted profiles in Figures S6, the full kinetic model provides a good description of the reaction behaviour in both THF and toluene over a variety of experimental conditions. However, although the involvement of the later intermediates is chemically reasonable, and the participation of ylides such as **Y2** and **Y4** is supported by independent experiments (e.g. trapping and crossover experiments), these intermediates are not directly observed during the in situ monitoring of the experiments because of the low steady-state concentrations. Consistent with this, the mass balance in the reaction profiles is accounted for predominantly by starting materials **1** and **2** and product **3**, with little detectable contribution from intermediate species.

The lack of the experimental data reference for these intermediates led to the less reliable fitted kinetic parameters. By contrast, these later steps are faster than formation of the ylide **Y2**, which is the turnover-limiting step of the reaction, and therefore have less influence on the overall temporal concentration profiles. Forcing a global fit with multiple poorly constrained late-stage parameters would therefore risk overfitting. To obtain a more robust and chemically meaningful description of the system, a simplified model was then used (see Scheme S1).

For reactions in THF, the simplified treatment also included precipitation/dissolution of **1·HCl**. This was implemented using a linear driving-force mass-transfer term with an effectively large mass-transfer coefficient, k_{LDF}, so that the dissolved concentration of **[1·HCl]** does not exceed its maximum solubility, c*. In practice, this treatment enforces effectively immediate dissolution and precipitation of **1·HCl** (equations S(3) and S(4)). The full simplified model, ordinary differential equation (ODE) system, and fitted parameters are given in Scheme S1, and this simplified model affords an overall better fit to the experimental data (see Figure S7).

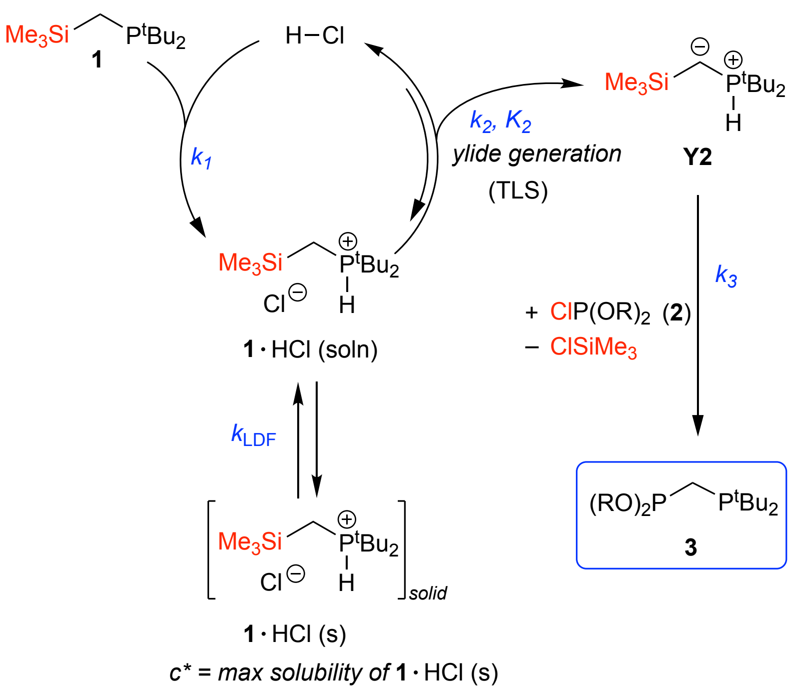

Scheme S1 Kinetic model and the derived ODE system for the simplified proposed mechanism in each solvent below. c^* is the solubility of $\mathbf{1} \cdot \mathrm{HCl}$, k_{LDF} is the linear-driving force modelled mass-transfer coefficient, and Vol is the volume of the monitored sample to calibrate the mass unit.

(A) Reactions in THF (with mass-transfer to assume the saturation of $\mathbf{1} \cdot \mathrm{HCl}$):

$$\mathbf{1} + \mathrm{HCl} \rightarrow \mathbf{1} \cdot \mathrm{HCl}; \quad k_1 = 3.66\ (\pm\ 0.3) \times 10^1\ \mathrm{M^{-1}s^{-1}}$$

$$\mathbf{1} \cdot \mathrm{HCl} \leftrightarrow \mathbf{1} \cdot \mathrm{HCl_s}; \quad k_{LDF} \geq 1 \times 10^3\ \mathrm{s^{-1}}; \quad c^* = 1.9\ (\pm\ 0.1) \times\ 10^{-4}\ \mathrm{M}$$

$$\mathbf{1} \cdot \mathrm{HCl} \leftrightarrow \mathbf{Y2}\ + \mathrm{HCl}\,; \quad k_2 = 5.35\ (\pm\ 0.05) \times 10^{-1}\ \mathrm{M^{-1}s^{-1}}; \ K_2 = 8.83\ (\pm\ 0.8) \times 10^{-5}\ \mathrm{M}$$

$$\mathbf{Y2} + \mathbf{2} \rightarrow \mathbf{3} + \mathrm{TMSCl}\,; \quad k_3 = 1.04\ (\pm\ 0.07) \times 10^2\ \mathrm{M^{-1}s^{-1}}$$

(B) Reactions in toluene:

$$\mathbf{1} + \mathrm{HCl} \rightarrow \mathbf{1} \cdot \mathrm{HCl}; \quad k_1 = 1.09\ (\pm\ 0.2) \times 10^2\ \mathrm{M^{-1}s^{-1}}$$

$$\mathbf{1} \cdot \mathrm{HCl} \leftrightarrow \mathbf{Y2}\ + \mathrm{HCl}\,; \quad k_2 = 2.87\ (\pm\ 0.05) \times 10^{-1}\ \mathrm{M^{-1}s^{-1}}; \ K_2 = 6.87\ (\pm\ 1) \times 10^{-6}\ \mathrm{M}$$

$$\mathbf{Y2} + \mathbf{2} \rightarrow \mathbf{3} + \mathrm{TMSCl}\,; \quad k_3 = 2.72\ (\pm\ 0.5)\ \mathrm{M^{-1}s^{-1}}$$

(A) ODEs in THF (with mass-transfer, controlled by the conditional function $\mathrm{X(s)}$):

$$(S1)\quad \frac{d[\mathbf{1}]}{dt} = -k_1[\mathbf{1}][\mathrm{HCl}]$$

$$(S2)\quad \frac{\mathrm{d[HCl]}}{\mathrm{dt}} = -k_1[\mathbf{1}][\mathrm{HCl}] + k_2[\mathbf{1}\cdot\mathrm{HCl}] - \frac{k_2}{K_2}[\mathbf{Y2}][\mathrm{HCl}]$$

$$(S3)\quad \frac{d[\mathbf{1}\cdot\mathrm{HCl}]}{dt} = +k_1[\mathbf{1}][\mathrm{HCl}] - k_2[\mathbf{1}\cdot\mathrm{HCl}] + \frac{k_2}{K_2}[\mathbf{Y2}][\mathrm{HCl}] + k_{LA}(c^* - [\mathbf{1}\cdot\mathrm{HCl}])\cdot\mathrm{X(s)};$$

$$\mathrm{X(s)} = \begin{cases} 0, & [\mathbf{1}\cdot\mathrm{HCl_s}] = 0 \\ 1, & [\mathbf{1}\cdot\mathrm{HCl_s}] > 0 \end{cases}$$

$$(S4)\quad \frac{d[\mathbf{1}\cdot\mathrm{HCl_s}]}{dt} = -Vol \times k_{LDF}(c^* - [\mathbf{1}\cdot\mathrm{HCl}])$$

$$(S5)\quad \frac{d[\mathbf{Y2}]}{dt} = +k_2[\mathbf{1}\cdot\mathrm{HCl}] - \frac{k_2}{K_2}[\mathbf{Y2}][\mathrm{HCl}] - k_3[\mathbf{Y2}][\mathbf{2}]$$

$$(S6)\quad \frac{d[\mathbf{2}]}{dt} = -k_3[\mathbf{Y2}][\mathbf{2}]$$

$$(S7)\quad \frac{d[\mathbf{3}]}{dt} = +k_3[\mathbf{Y2}][\mathbf{2}]$$

$$(S8)\quad \frac{d[TMSCl]}{dt} = +k_3[\mathbf{Y2}][\mathbf{2}]$$

(B) ODEs in toluene:

$$(S1')\quad \frac{d[\mathbf{1}]}{dt} = -k_1[\mathbf{1}][\mathrm{HCl}]$$

$$(S2')\quad \frac{\mathrm{d[HCl]}}{\mathrm{dt}} = -k_1[\mathbf{1}][\mathrm{HCl}] + k_2[\mathbf{1}\cdot\mathrm{HCl}] - \frac{k_2}{K_2}[\mathbf{Y2}][\mathrm{HCl}]$$

$$(S3')\quad \frac{d[\mathbf{1}\cdot\mathrm{HCl}]}{dt} = +k_1[\mathbf{1}][\mathrm{HCl}] - k_2[\mathbf{1}\cdot\mathrm{HCl}] + \frac{k_2}{K_2}[\mathbf{Y2}][\mathrm{HCl}]$$

$$(S5')\quad \frac{d[\mathbf{Y2}]}{dt} = +k_2[\mathbf{1}\cdot\mathrm{HCl}] - \frac{k_2}{K_2}[\mathbf{Y2}][\mathrm{HCl}] - k_3[\mathbf{Y2}][\mathbf{2}]$$

$$(S6')\quad \frac{d[\mathbf{2}]}{dt} = -k_3[\mathbf{Y2}][\mathbf{2}]$$

$$(S7')\quad \frac{d[\mathbf{3}]}{dt} = +k_3[\mathbf{Y2}][\mathbf{2}]$$

$$(S8')\quad \frac{d[TMSCl]}{dt} = +k_3[\mathbf{Y2}][\mathbf{2}]$$

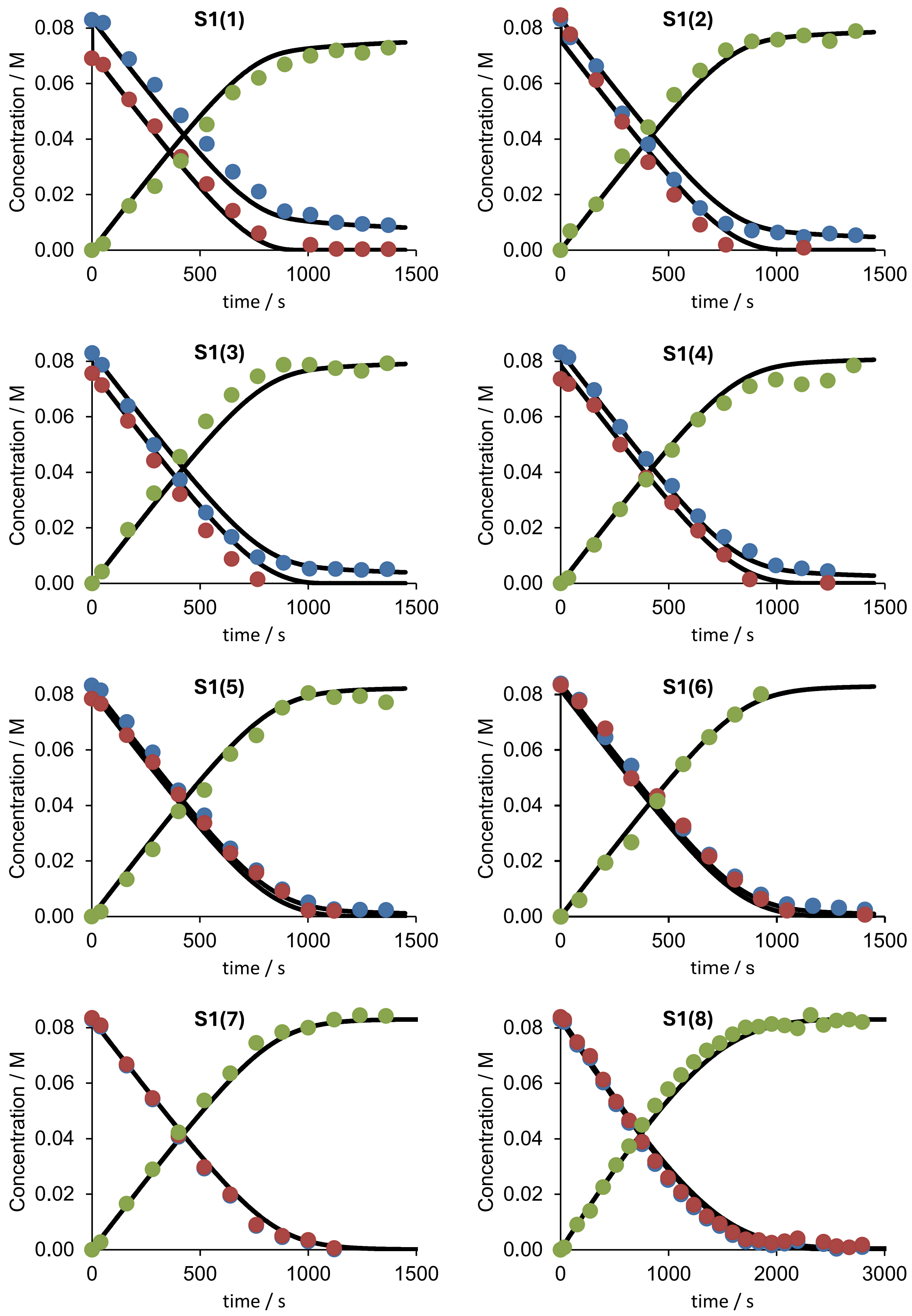

Figure S7: Reaction profiles for the conversion of **1** (red) and **2** (blue) to **3** (green), simulated with the model of the simplified mechanism (**Scheme S1**). Initial concentrations were taken from **Tables S1** (entries S1(1)–S1(9) in THF) and **S2** (entries S2(1)–S2(7) in toluene) at 40 °C. Circles represent the experimental data obtained by integration of the $^{31}P\{^1H\}$ NMR spectra, and solid lines represent the simulated data generated using the parameters listed in **Scheme S1**. The R^2 values for all three profiles are > 0.98. **(continued below)**

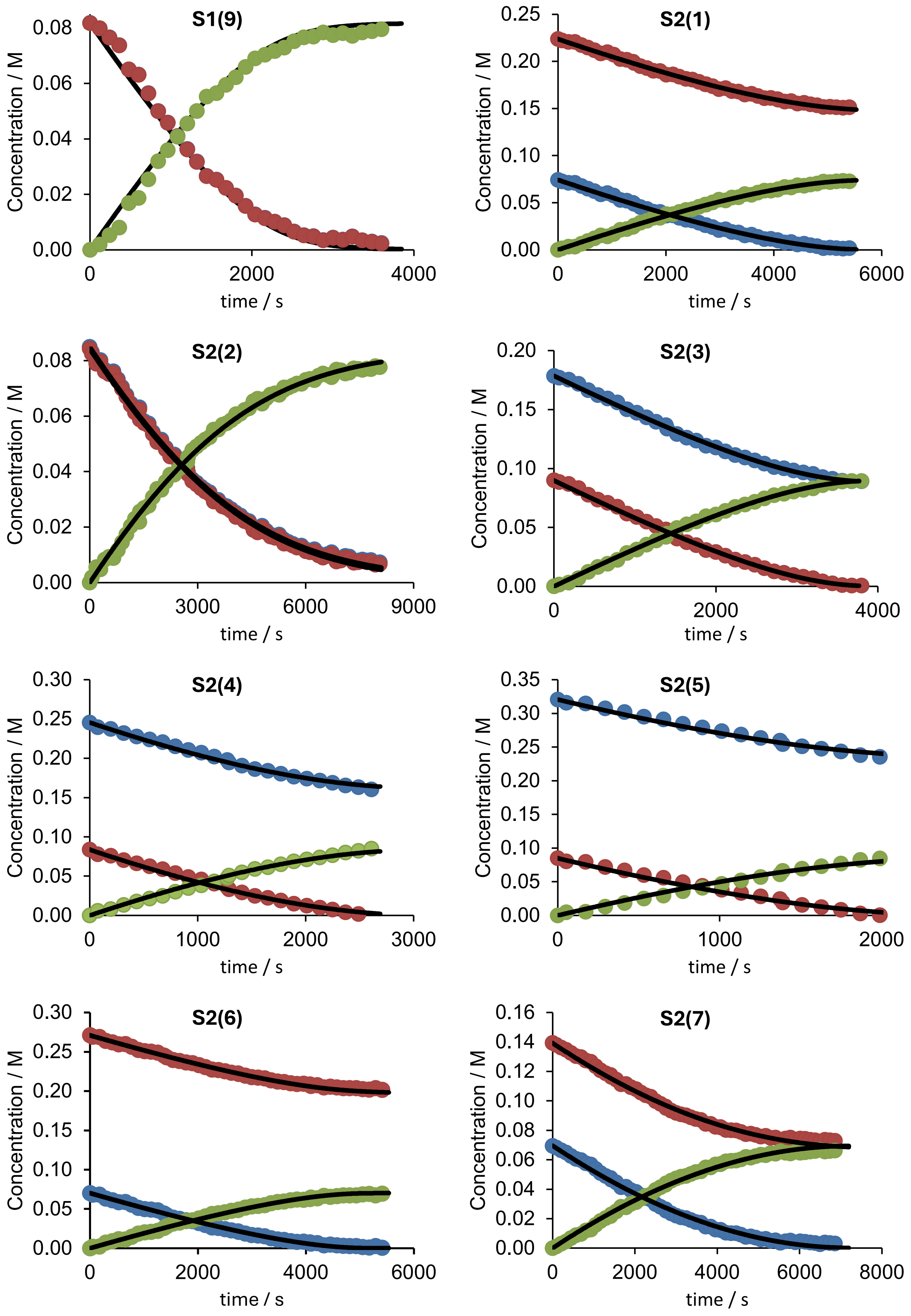

Figure S7: Reaction profiles for the conversion of **1** (red) and **2** (blue) to **3** (green), simulated with the model of the simplified mechanism (Scheme S1). Initial concentrations were taken from Tables S1 (entries S1(1)–S1(9) in THF) and S2 (entries S2(1)–S2(7) in toluene) at 40 °C. Circles represent the experimental data obtained by integration of the $^{31}P\{^{1}H\}$ NMR spectra, and solid lines represent the simulated data generated using the parameters listed in Scheme S1. The R^2 values for all three profiles are > 0.98.

S4 NMR study of the chlorodesilylation of $^tBu_2PCH_2SiMe_3$ with DCl

Under Ar, a solution of **1** (10 mg, 0.040 mmol) in THF (0.50 mL) was treated with a THF/Et_2O solution of DCl (0.50 mL, 0.080 M, 0.040 mmol) and the mixture shaken and then left to stand. The ^{31}P NMR spectrum, recorded after 24 and 72 h showed that there was no remaining **1** after 72 h and that 2 major products ($^tBu_2PCH_2D$ and $^tBu_2PCH_3$) were formed along with a small amount (ca. 1%) of a minor product tentatively assigned to $^tBu_2PCHD_2$ based on its δ_P value (Figure S8). The assignments of the major products were confirmed by the ^1H NMR spectrum obtained with solvent suppression (Figure S9).

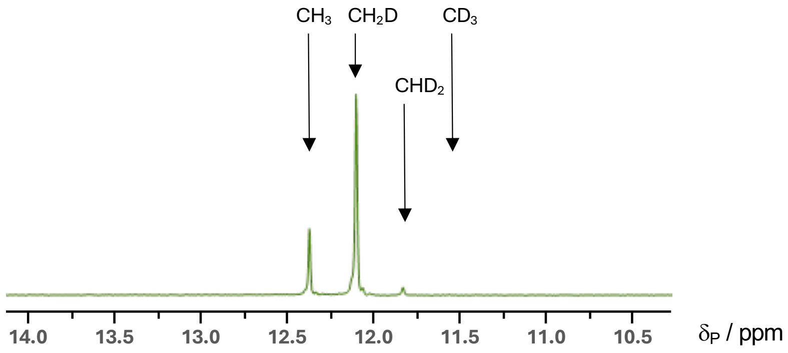

Figure S8: $^{31}P\{^1H\}$ NMR spectrum of the product obtained from treatment of **1** with 1 equiv. of DCl in THF after 24 h. The shifts of $^tBu_2PCD_xH_{3-x}$, x = 0, 1, 3, (arrows) are assigned from independently prepared reference samples and the minor peak at δ_P 11.8 is assigned to $^tBu_2PCHD_2$ based on the additivity of the $^2\Delta P(D)$ isotope shifts.

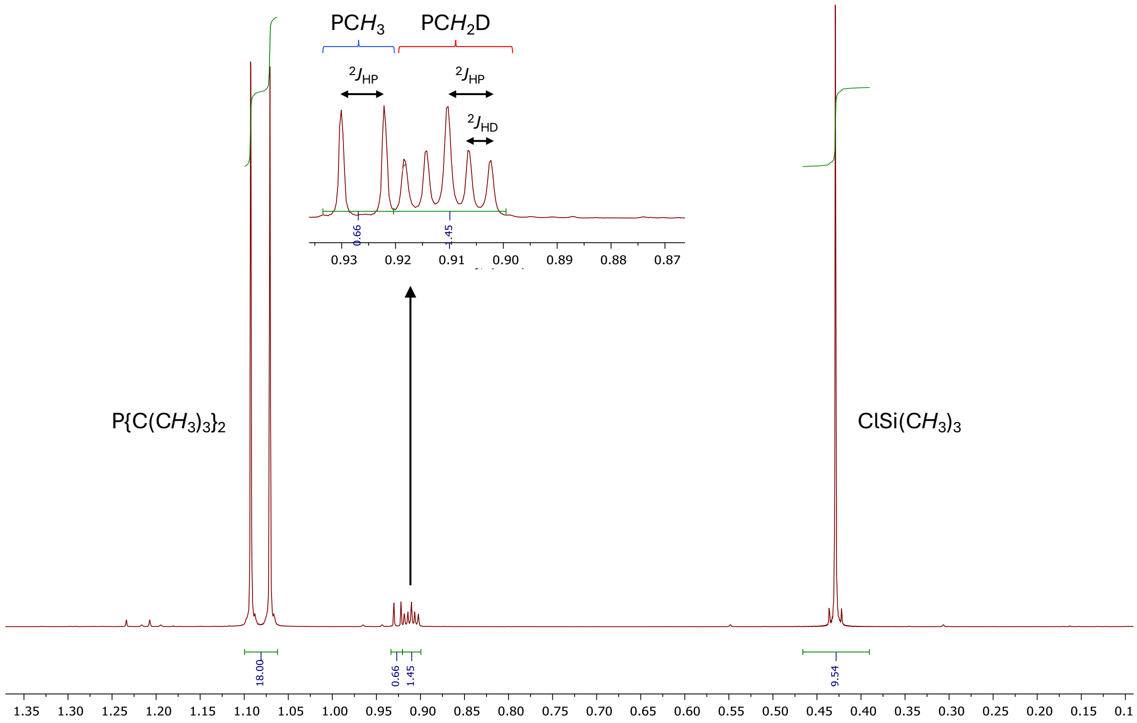

Figure S9: ^{1}H NMR spectrum of the product obtained from treatment of **1** with 1 equiv. of DCl. The ^{2}H NMR spectrum showed a broad signal at 0.90 ppm.

S5 Preparation of $^tBu_2PCH_2CHAr(OSiMe_3)$ (IV, Ar = *o*-C_6H_4F) via a Peterson-like reaction

A solution of **1** (25 mg, 0.10 mmol) in THF (1 mL) was added to a solution of 2-fluorobenzaldehyde (14 mg, 0.11 mmol) in THF (1 mL) and the mixture stirred at ambient temperature for 16 h. The volatiles were then removed under reduced pressure to give **IV** as a colorless oil which was characterised by NMR spectroscopy only. $^{31}P\{^1H\}$ NMR (122 MHz, CD_2Cl_2): δ 19.60 (d, $^5J_{PF}$ = 11.2 Hz). $^{19}F\{^1H\}$ (282 MHz, CD_2Cl_2): δ −119.60 (d, $^5J_{PF}$ = 11.2 Hz). ^{1}H NMR (300 MHz, CD_2Cl_2): δ 0.05 (9H, s, $SiMe_3$); 1.05 (9H, d, $^3J_{PH}$ = 10.7 Hz, CMe_3); 1.13 (9H, d, $^3J_{PH}$ = 10.9 Hz, CMe_3); 1.84 (2H, m, CH_2); 5.09 (1H, apparent q, J = 6.7 Hz, CHO); 6.99 (1H, m, ArH); 7.10-7.27 (2H, m, ArH); 7.51 (1H, m, ArH).

S6 Crossover experiments

(1) A solution of **3** (10 mg, 0.021 mmol) in THF (0.5 mL) was added to a solution of PCl(3,3'-diphenyl-2,2'-binaphthyldiolate), **2a** (11 mg, 0.022 mmol) in THF (0.5mL) in a Young's NMR tube and the mixture shaken vigorously for 2 min; no immediate reaction was apparent by $^{31}P\{^1H\}$ NMR spectrum spectroscopy after 10 min but after 16 h, the $^{31}P\{^1H\}$ NMR spectrum shown in Figure S10 was obtained. This spectrum is consistent with a mixture of **3** and $^tBu_2PCH_2P$(3,3'-diphenyl-2,2'-binaphthyldiolate) (**3a**) as well as chlorophosphites **2** and **2a** in the ratios indicated in Figure S10.

(2) A solution of **3a** (13 mg, 0.021 mmol) in THF (0.5 mL) was added to a solution of **2** (7 mg, 0.020 mmol) in THF (0.5 mL) in a Young's NMR tube and the mixture shaken vigorously for 2 min. After 16 h, a $^{31}P\{^1H\}$ NMR spectrum very similar to that shown in Figure S10 was obtained consistent with the measured proportions of **3** / **3a** and **2** / **2a** correspond to an equilibrium mixture.

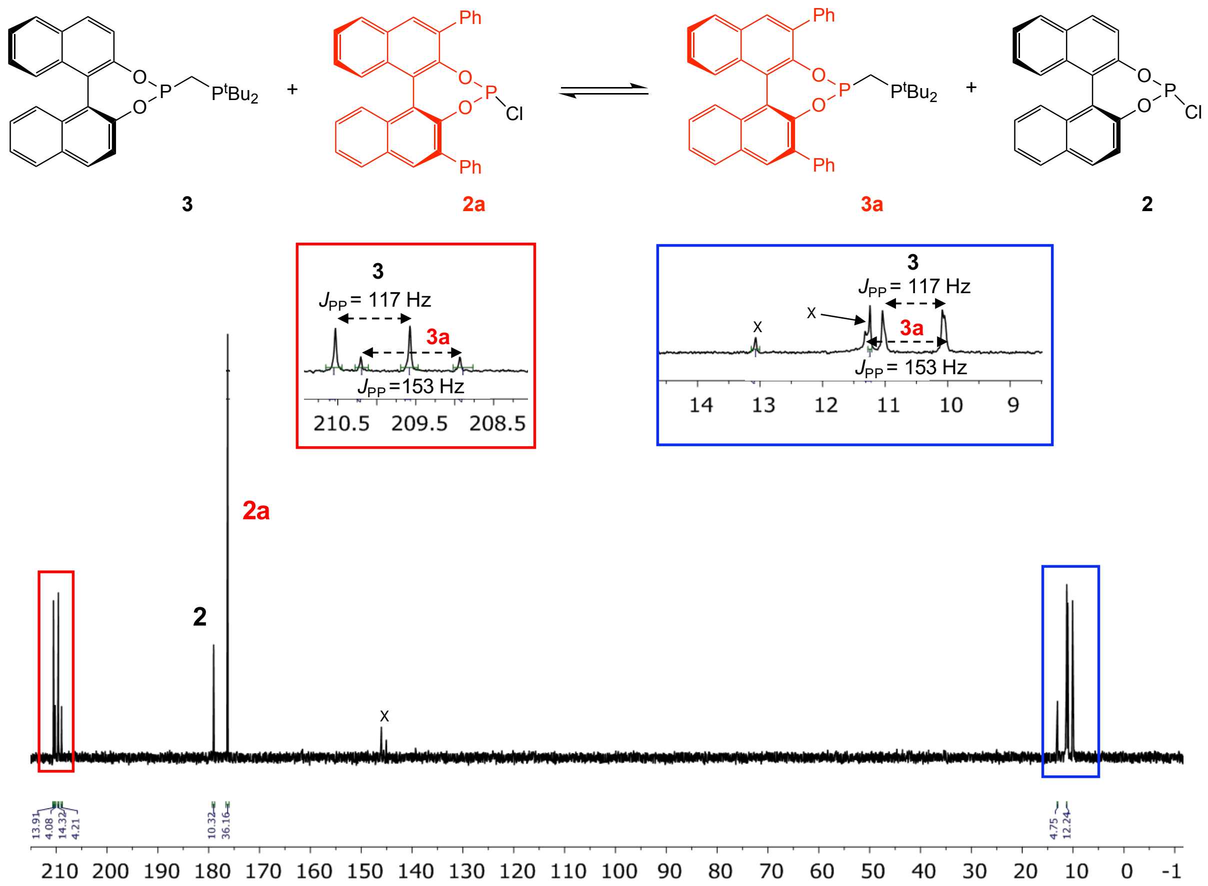

Figure S10: $^{31}P\{^1H\}$ NMR spectrum of the mixture obtained 16 h after mixing THF solutions of **3** and **2a**. A very similar mixture was obtained 16 h after mixing THF solutions of **3a** and **2** indicating that this represents the equilibrium mixture. Peaks labelled 'x' are unidentified impurities.

S7 NMR study of the reaction between [$^tBu_2P(D)CH_2SiMe_3$]Cl (1·DCl) and 2

A solution of PCl(1,1'-bi-2-naphtholate) (**2**) (20 mg, 0.057 mmol) in THF (0.5 mL) was added to **1**·DCl (15 mg, 0.056 mmol) in THF (0.5 mL) and the mixture shaken and then set aside for 24 h. The $^{31}P\{^1H\}$ NMR spectrum of the product (Figure S11) was interpreted as showing a mixture of isotopologues $^tBu_2PCD_xH_{2-x}P$(1,1'-bi-2-naphtholate) where x = 0, 1 and 2. The volatiles were removed and the product redissolved in $CDCl_3$ to obtain the ^1H NMR spectrum (Figure S12) which showed a multiplet at δ 1.9 for the methylene group.

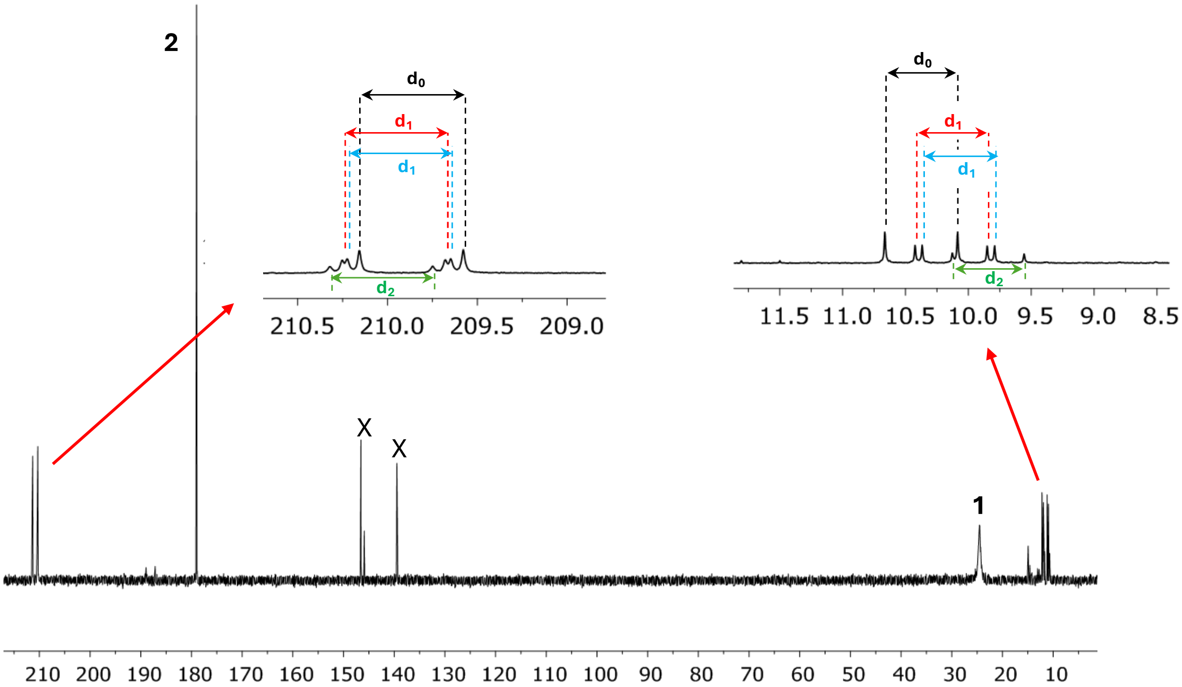

Figure S11: $^{31}P\{^{1}H\}$ NMR spectrum of the mixture of isotopologues of **3** formed by the reaction of **2** with **1**·DCl labelled d_0, d_1 and d_2 corresponding to the number of D atoms incorporated. Note that for the d_1 compound, two diastereoisomers are identified, as expected due to the combination of the dissymmetric 1,1'-bi-2-naphthol moiety and the stereogenic CHD group. Signals marked X are unassigned impurities.

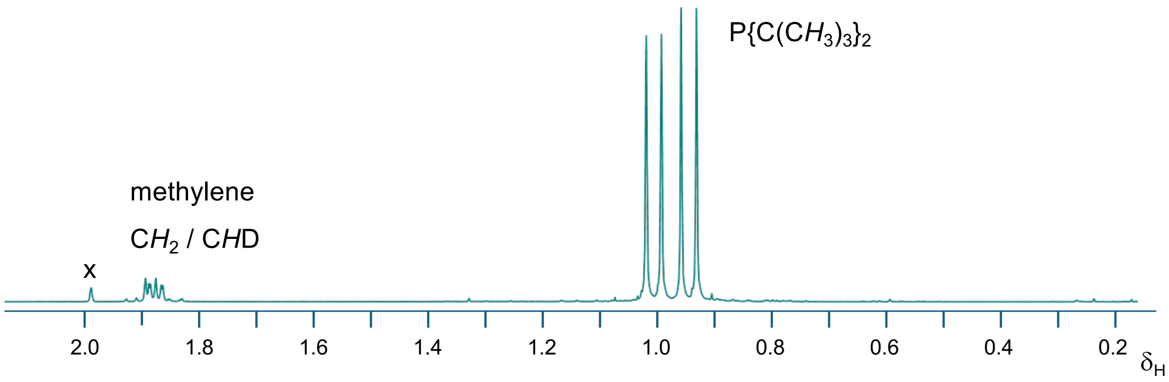

Figure S12: ^{1}H NMR spectrum of the mixture of isotopologues of **3**. The two 1:1 doublets at *ca.* 1.0 ppm are for the diastereotopic t-Bu groups showing $^{3}J_{HP}$ coupling. The methylene signal near 1.9 ppm is tentatively assigned to a sharp doublet for the CH_2 and a broad doublet assigned to the CHD, with both signals showing similar values of $^{2}J_{HP}$; the broadness of the CHD signal is attributed to unresolved $^{2}J_{HD}$ coupling and there being two diastereoisomers present. The signal marked X is an unassigned impurity. A ^{2}H NMR spectrum of this mixture showed a broad unresolved peak, also at δ 1.9.

1 Pangborn, A. B.; Giardello, M. A.; Grubbs, R. H.; Rosen, R. K.; Timmers, F. J. Safe and convenient procedure for solvent purification. *Organometallics*, **1996**, *15*, 1518-1520.

2 Hazeland, E. L.; Chapman, A. M.; Pringle, P. G.; Sparkes, H. A. A one-step, modular route to optically-active diphos ligands. *Chem. Commun*., **2015**, *51*, 10206-10209.
